# Mechanisms of Na^+^ uptake from freshwater habitats in animals

**DOI:** 10.3389/fphys.2022.1006113

**Published:** 2022-10-18

**Authors:** Carol Eunmi Lee, Guy Charmantier, Catherine Lorin-Nebel

**Affiliations:** ^1^ Department of Integrative Biology, University of Wisconsin, Madison, WI, United States; ^2^ MARBEC, Univ Montpellier, CNRS, Ifremer, IRD, Montpellier, France

**Keywords:** osmoregulation, ionic regulation, Arthropoda, fish, V-type H^+^-ATPase, Na^+^/K^+^-ATPase, Na^+^/H^+^-antiporter

## Abstract

Life in fresh water is osmotically and energetically challenging for living organisms, requiring increases in ion uptake from dilute environments. However, mechanisms of ion uptake from freshwater environments are still poorly understood and controversial, especially in arthropods, for which several hypothetical models have been proposed based on incomplete data. One compelling model involves the proton pump V-type H^+^ ATPase (VHA), which energizes the apical membrane, enabling the uptake of Na^+^ (and other cations) *via* an unknown Na^+^ transporter (referred to as the “Wieczorek Exchanger” in insects). What evidence exists for this model of ion uptake and what is this mystery exchanger or channel that cooperates with VHA? We present results from studies that explore this question in crustaceans, insects, and teleost fish. We argue that the Na^+^/H^+^ antiporter (NHA) is a likely candidate for the Wieczorek Exchanger in many crustaceans and insects; although, there is no evidence that this is the case for fish. NHA was discovered relatively recently in animals and its functions have not been well characterized. Teleost fish exhibit redundancy of Na^+^ uptake pathways at the gill level, performed by different ion transporter paralogs in diverse cell types, apparently enabling tolerance of low environmental salinity and various pH levels. We argue that much more research is needed on overall mechanisms of ion uptake from freshwater habitats, especially on NHA and other potential Wieczorek Exchangers. Such insights gained would contribute greatly to our general understanding of ionic regulation in diverse species across habitats.

## Introduction: The problem of ion uptake from fresh water

Marine to freshwater colonizations represent among the most dramatic evolutionary transitions in the history of life (Hutchinson, 1957; Little, 1983, 1990; Miller and Labandeira, 2002). Most animals evolved in the sea, and of the ∼35 animal phyla, only 16 phyla contain representatives that have colonized freshwater habitats during the course of evolutionary history (Hutchinson, 1957; Little, 1983; Little, 1990; Lee and Bell, 1999; Miller and Labandeira, 2002). Marine and many estuarine animals, aside from most vertebrates, tend to possess body fluids that resemble the surrounding seawater in ionic composition (Willmer et al., 2008). In contrast, freshwater animals tend to constantly lose their ions passively, mainly through their body surface or gills. Thus, active ion transport and the tightening of epithelia are essential to compensate for passive ionic losses across epithelia that are in direct or indirect contact with the freshwater environment.

Thus, living in dilute environments poses great challenges for acquiring essential ions against steep concentration gradients between body fluids and the environment (Beyenbach, 2001; Morris, 2001; Tsai and Lin, 2007; Lee et al., 2012). Freshwater animals cannot survive without maintaining elevated extracellular fluid (hemolymph or blood) osmolalities relative to the very dilute environment. In general, invertebrates tend to maintain a broader range of hemolymph osmolalities and often osmoconform to a relatively wide range of intermediate salinities. In contrast, teleost fish are strong regulators that maintain a much narrower range of blood osmotic concentrations.

For instance, invertebrates generally maintain a broad range of extracellular osmolalities in fresh water ranging from ca. 200 to 400 mOsm.kg^−1^, with values as high as 600 mOsm.kg^−1^ in the Chinese mitten crab *Eriocheir sinensis* and as low as 50 mOsm.kg^−1^ in some mollusk species (Schmidt-Nieslen, 1997; Willmer et al., 2008; Charmantier et al., 2009; Evans and Claiborne, 2009). In contrast, teleost fish maintain more constant blood osmolalities, from around 260 to 380 mOsm.kg^−1^ (Evans and Claiborne, 2009), with freshwater fish having lower blood osmolalities (i.e., 260 mOsm/kg^−1^ in carps *Cyprinus carpio*; Holmes and Donaldson, 1969) than marine species (i.e., 360–380 mOsm.kg^−1^ in the European sea bass *Dicentrarchus labrax;* L’Honoré et al., 2019). For fish under freshwater conditions, blood osmolality is generally maintained far above 250 mOsm.kg^−1^, except in fish that are stressed or intolerant of fresh water (L’Honoré et al., 2019).

Overcoming the challenges of living in ion-poor environments through the evolution of body fluid regulation was critical for freshwater colonizations, which then provided key adaptations for the colonization of land (Wolcott, 1992; Anger, 2001; Morris, 2001; Glenner et al., 2006). Yet, basic questions regarding evolutionary adaptations during saline to freshwater transitions remain unresolved (Charmantier et al., 2009; Evans and Claiborne, 2009; Hwang et al., 2011; Dymowska et al., 2012). In particular, fundamental mechanisms of ionic regulation remain incompletely understood in most invertebrates, especially regarding ion uptake from very low salinities (Charmantier et al., 2009; McNamara and Faria, 2012). In teleost fish, ion uptake mechanisms are very diverse, possibly due to major evolutionary changes in genome architecture and diversification of ion transporter gene families (Desvignes et al., 2021). Numerous studies have described these diverse ion uptake mechanisms in fish, but only in a few species (Dymowska et al., 2012; Hwang and Chou, 2013; Zimmer and Perry, 2022).

**FIGURE 1:**
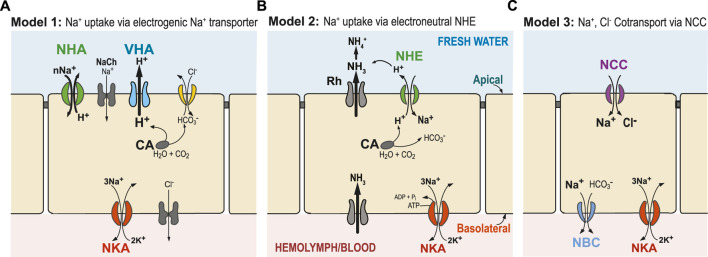
Generalized hypothetical models of ion uptake by ionocytes in aquatic animals under freshwater conditions. **(A)** Model 1 (Wieczorek’s Model): VHA (blue) pumps out H^+^ and creates an electrical gradient, through which Na^+^ is transported into the cell through an electrogenic Na^+^ transporter (potentially NHA or Na^+^ channel, NaCh). **(B)** Model 2: Ammonia is transported out of the cell by an ammonia transporter (Rh protein), which then drives electroneutral NHE (green) to export H^+^, and consequently import Na^+^. **(C)** Model 3: Na^+^ and Cl^−^ are co-transported to the cell by the NCC or NCC-like cotransporter. In these three models, transport of Na^+^ across the basolateral membrane from the cell to the hemolymph or blood is performed by the primary transporter NKA (red) and, potentially, also by NBC (blue, in Model 3). Cytosolic carbonic anhydrase (CA) performs CO_2_ hydration, supplying H^+^ and HCO_3_^−^ to apical or basolateral ion transporters (Henry 1996). Additionally, chloride uptake might occur through an Cl^−^/HCO_3_^−^ exchanger (yellow) or some other bicarbonate exchanger. Alternative models have also been proposed, and not all relevant ion transporters are shown.

A key toward understanding ion uptake mechanisms likely resides in the functions of *ionocytes* (Evans et al., 2005; Freire et al., 2008; Charmantier et al., 2009; Hwang and Perry, 2010; Hiroi and McCormick, 2012). Ionocytes, formerly called chloride cells or mitochondrion-rich cells (in fish gills), are cells rich in mitochondria and specialized for ion transport. These specialized cells perform ion uptake or excretion to regulate body fluid concentrations. These cells are ubiquitous across a wide range of osmoregulatory organs, including in crustacean gills, crustacean antennal and maxillary glands, insect Malpighian tubules and alimentary canal, fish gills, kidney, and intestine, as well as skin of fish larvae and embryos (Piermarini and Evans, 2000; Weihrauch et al., 2004; Varsamos et al., 2005; Patrick et al., 2006; Charmantier et al., 2009; Hiroi and McCormick, 2012). These cells possess deep basolateral infoldings and a suite of ion transporters and channels on their apical (outer) and basolateral (inner) membranes (Figure 1). Thus, the functioning of ion transporters and channels, and their cooperation within ionocytes, play critical roles for colonizations and migrations between different habitats. Yet, how ionocytes take up ions from environments is still not fully resolved in invertebrates, particularly from freshwater environments (Charmantier et al., 2009; McNamara and Faria, 2012). In fish, ion uptake mechanisms are well understood in only a few species, notably in their gills or embryonic skin (Dymowska et al., 2012; Kumai and Perry, 2012; Guh et al., 2015), and need to be investigated in other ecologically relevant species.

In addition to the functions of ionocytes, permeability between them (paracellular permeability) is crucial to consider when addressing ion transport. In freshwater osmoregulatory epithelia, intercellular adhesion complexes known as tight junctions control paracellular diffusion of ions and water (Tipsmark et al., 2008; Kolosov et al., 2013). While this mechanism of ionic regulation is important, this topic is beyond the scope of this particular paper.

In terms of driving ion uptake within ionocytes, Na^+^/K^+^-ATPase (NKA) was initially thought to provide the major limiting energetic driving force for ion uptake across all types of salinity environments (Towle, 1984). NKA was first shown to be basolaterally localized in fish gill ionocytes in the 1970s (Karnaky et al., 1976). NKA pumps Na^+^ from the cytosol to the hemolymph in exchange for K^+^ transported into the cell. The resulting accumulation of K^+^ in the cytoplasm results in diffusion of K^+^ back to hemolymph through basolateral K^+^ channels or apical K^+^ secretion (reviews in Kirschner, 2004; McNamara and Faria, 2012; Horng et al., 2017; Leone et al., 2017). As three Na^+^ ions are exchanged for two K^+^ ions, the cytosol becomes electronegative. NKA activity thus results in establishing and maintaining two gradients, a concentration gradient and an electrical gradient, both driving apical entry of Na^+^ from the external medium into the cell. However, based on thermodynamic principles, NKA is insufficient to drive ion uptake below NaCl concentrations of ∼1.0 mM (Larsen et al., 1996).

Thus, under very low salinity conditions, an additional energizing ion transporter is required. Since the 1990s, evidence has been mounting that an apically localized proton pump V-type H^+^-ATPase (VHA) plays a crucial role in energizing ion uptake in low salinity environments (and from urine of terrestrial organisms) (Figure 1A, Model 1) (Lin and Randall, 1991). The role of an electrogenic proton pump in driving sodium uptake was originally discovered in frog skin (Ehrenfeld et al., 1985), and then subsequently hypothesized by Wieczorek et al. in insects (Wieczorek et al., 1991; Wieczorek et al., 1999; Chambrey et al., 2013) and by Avella and Bornancin in teleost fish (rainbow trout *Oncorhynchus mykiss*) (Avella and Bornancin, 1989). VHA localized on the apical (outer) membrane of the cell could generate an electrochemical potential by pumping H^+^ out of the cell. This electrical gradient could then be used to take up ions, such as Na^+^, *via* secondary transporters or channels. However, the identities of these secondary transporters responsible for Na^+^ uptake have been unclear and controversial (see next sections; Charmantier et al., 2009; Evans and Claiborne, 2009; Kumai and Perry, 2012; McNamara and Faria, 2012).

Several secondary transporters and channels have been hypothesized to cooperate with VHA to transport Na^+^ into ionocytes, such as a putative Na^+^ channel (McNamara and Faria, 2012), the Na^+^/H^+^ exchanger (NHE, SCL9A) (Claiborne et al., 1999; Edwards et al., 1999; Towle and Weihrauch, 2001), or the Na^+^/H^+^ antiporter (NHA, SLC9B) (Xiang et al., 2012; Posavi et al., 2020; Stern and Lee, 2020). However, evidence for these secondary transporters working with VHA has been relatively limited (but see Xiang et al., 2012; Dymowska et al., 2014; Dymowska et al., 2015). Based on stoichiometry, Wieczorek et al. other hypothesized that an electrogenic antiporter that exchanges cations with H^+^ must be cooperating with VHA (Wieczorek et al., 1991; Beyenbach and Wieczorek, 2006). This missing transporter had been dubbed the “Wieczorek exchanger” and its identity had remained a subject of debate.

This secondary Na^+^ transporter that cooperates with VHA would have to be electrogenic, meaning that ion uptake would involve a net charge translocation across the membrane (e.g., by NHA or Na^+^ channel, Figure 1A). For example, importing just Na^+^ ion or exchanging two Na^+^ for one H^+^ would be electrogenic, whereas exchanging one Na^+^ for one H^+^ would be electroneutral (e.g., NHE in Figure 1B). Utilizing the electrical gradient (positive charge outside) generated by apical VHA would drive the secondary transporter to perform cation uptake, such as by NHA (SLC9B) or Na^+^ channel (NaCh), and thus result in charge translocation. However, export of H^+^ by VHA would not drive electroneutral Na^+^ uptake from fresh water by NHE (SLC9A), because NHE exchange (of one H^+^ out for one Na^+^ in) would actually run against the H^+^ gradient (chemical gradient, ΔpH) generated by VHA (Potts, 1994).

Starting in the late 1980s, kinetic Na^+^ flux analyses suggested the presence of an electrogenic 2Na^+^/1H^+^ antiporter in invertebrates that could potentially act as Wieczorek exchangers; although, the genetic identity of these transporters had remained unknown (Ahearn and Clay, 1989; Shetlar and Towle, 1989; Ahearn and Pierette, 1991; Ahearn et al., 1994). More recently, a new ion transporter gene family was discovered for the first time in animals, identified as a putative electrogenic Na^+^/H^+^ antiporter (NHA or SLC9B, Figure 1A, green) and genetically distinct from the previously known electroneutral Na^+^/H^+^ exchanger (NHE or SLC9A, Figure 1B) (Brett et al., 2005; Rheault et al., 2007; Day et al., 2008; Xiang et al., 2012). These Na^+^/H^+^ antiporters (NHA), previously known in bacteria and yeast, were discovered and characterized in the fruit fly *Drosophila melanogaster* and mosquito *Anopheles gambiae* (Rheault et al., 2007; Day et al., 2008; Xiang et al., 2012). In particular, one type of NHA, found in apical membranes of larval mosquito Malpighian tubules, was analyzed in a heterologous yeast system and found to perform cation uptake in what appeared to be an electrogenic manner (nCations/1H^+^) (Xiang et al., 2012). While this result provided support for Model 1, the evidence was not conclusive. While NHA does seem to be critical for ion homeostasis and response to salt stress, functional studies in animals have yielded divergent results, suggesting that its functions might vary among NHA paralogs, cell types, tissues, and taxa (Day et al., 2008; Xiang et al., 2012; Chintapalli et al., 2015).

Additional support for Model 1 was found relatively recently in fish gills (rainbow trout *Oncorhynchus mykiss* and zebrafish *Danio rerio*), but with the sodium channel ASIC4 (Dymowska et al., 2014; Dymowska et al., 2015). Acid-sensing ion channels (ASICs) are close relatives of the tetrapod epithelial sodium channels (ENaC) (Kellenberger and Schild, 2002) and were first analyzed in zebrafish neurons (Chen et al., 2010). ASIC4 was then localized in gill ionocytes of trout and zebrafish and investigated as potential Na^+^ channels that would facilitate apical Na^+^ uptake in fish acclimated to low salinity and low pH conditions. The apical coexpression of VHA and ASIC4.2 in zebrafish ionocytes indicates the presence of a Na^+^ transporter coupled to VHA, supporting Model 1 in freshwater fish (Dymowska et al., 2014, 2015). At this point, no other evidence exists for ASIC4 expression in gills of other teleost fish species.

On the other hand, data linking ammonia excretion with Na^+^ uptake, and the failure to find an electrogenic Na^+^ transporter in several systems (e.g., particularly in fish), led to the proposal of a second model (Model 2, Figure 1B) (Wright and Wood, 2009; Dymowska et al., 2012; Ito et al., 2013). In this model, an electroneutral Na^+^/H^+^ exchanger (NHE) located on the apical membrane of ionocytes exchanges one Na^+^ for one H^+^. However, apical NHE cannot function adequately under neutral to low external pH (high H^+^) and low external Na^+^ (e.g., freshwater conditions), as this exchanger is driven by environmental and cellular concentration gradients of Na^+^ and H^+^ and not by membrane potential (Parks et al., 2008). Consequently, if NHE were functioning exclusively under completely freshwater conditions and pH < 8.0, it would work in the opposite direction, and Na^+^ loss would dominate. To overcome these thermodynamic constraints that prevent NHE from functioning at lower external pH, an ammonia transporter has been proposed to transport NH_3_ out of the cell to “trap” H^+^ outside the cell. This exported NH_3_ would react with H^+^ and produce NH_4_^+^. Then the lowered external H^+^ concentration would promote H^+^ export out of the cell and facilitate Na^+^ uptake through NHE activity (Wright and Wood, 2009; Wu et al., 2010; Shih et al., 2012). Incipient ideas for this model were first formulated in the 1930’s, when Krogh found that Na^+^ uptake is coupled to NH_4_ excretion, without knowledge of the ion transporters/channels involved (Krogh, 1937).

In a third model, NaCl absorption occurs through an apical Na^+^,Cl^−^ cotransporter (NCC -like, also called NCC2, SLC12A10) (Model 3, Figure 1C). This hypothesized model is based on mammalian kidney distal tubule cells, where the Na^+^,Cl^-^ cotransporter (NCC, SLC12A3) is the major route promoting Na^+^ absorption (Plotkin et al., 1996; Yang et al., 1996; Meneton et al., 2000). The fact that apical NCC promotes NaCl uptake in a specific cell type, the NCC cell, is now well-established in several fish species (Hiroi et al., 2008; Wang et al., 2009; Dymowska et al., 2012). Thus far, basolateral NKA is the driving force known to typically facilitate apical NCC2-mediated NaCl transport.

At extremely low salinities, Model 1 is considered more likely to operate than Model 2, as the transmembrane voltage gradient generated by VHA could enable cations to be taken up from extremely low concentrations (Figure 1A). In Model 2, NHE could overcome its aforementioned thermodynamic constraints at extremely low salinities and lower pH (Dymowska et al., 2015) when ion uptake is coupled with the action of Rh protein (Figure 1B). How Model 3 is operating under low ionic concentrations remains an open question. It is possible that each ion uptake mechanism might operate under different salinities and pH levels, with Model 1 more favored under very low salinities and low pH (Dymowska et al., 2014; Dymowska et al., 2015). The three models (Figure 1) do not represent all the mechanisms that have been proposed for Na^+^ uptake, but they do represent plausible mechanisms, given the data (see next sections).

So, what is the current evidence that these mechanisms of ion uptake might be operating in animals residing in freshwater habitats? The next sections discuss the evidence for the three models described above (Figure 1) for selected taxa, namely, crustaceans, insects, and teleost fish. Members of these groups have served as the main models for exploring mechanisms of ion transport in aquatic habitats. Crustaceans and insects both belong to the arthropod subphylum Pancrustacea, with insects and related groups (hexapods) nested within the crustacean clade (Misof et al., 2014; Lozano-Fernandez et al., 2016). Thus, insects are essentially a lineage of crustaceans that have colonized land (Regier et al., 2005; Glenner et al., 2006). Fish are interesting examples of highly regulated systems with respect to osmotic and ionic regulation, where their blood osmolality can be highly regulated regardless of the surrounding media (achieving greater homeostasis).

A key point to mention here is that ionocytes perform multiple functions, which include acid-based regulation and ion excretion, such as ammonia excretion, as well ion uptake. While this paper focuses on ion uptake from fresh water, it is important to note that ion uptake, acid-base regulation, and excretion of ammonia are inextricably linked (see reviews by Krogh, 1939; Evans, 2009; Evans, 2011, Hwang et al., 2011; Guh et al., 2015). For instance, ion uptake often occurs simultaneously with acid-base regulation and frequently involves the same ion transporters, such as VHA, Na^+^/H^+^ antiporter (NHA), Na^+^/H^+^ exchanger (NHE), Na^+^,HCO_3_^−^ cotransporter (NBC), and Cl^−^/HCO_3_^−^ exchanger (AE or some other anion exchanger). However, discussions of mechanisms of acid-base regulation are beyond the scope of this review.

This review is the first to explicitly discuss the evidence for the main proposed models of ion uptake in freshwater habitats (Figure 1) in a wide range of taxa. While these three models have been discussed in many studies, this study attempts to compare and contrast ion uptake mechanisms across three main groups (crustaceans, insects, and teleost fish), and also highlight gaps in our understanding that should be examined in future studies. This paper focuses on these three groups because most aquatic physiological studies have focused on model systems belonging to these groups. Research on many of these systems is still nascent and our knowledge is still incomplete and often without consensus. Nevertheless, we hope that this review serves as a useful reference for what is currently known regarding models of ion uptake (particularly sodium uptake) in freshwater habitats.

## Ion uptake in crustaceans

The origin of the first “crustaceans” dates to the Lower Cambrian, more than 500 million years ago (Schram, 1982; Chen et al., 2001; Glenner et al., 2006; Lozano-Fernandez et al., 2016). Members of the paraphyletic crustacean clade have colonized a wide variety of habitats and exhibit a wide array of osmoregulatory patterns, from osmoconformers to strong osmoregulators. Among the ca. 67000 extant described species (Zhang et al., 2011), most live in aquatic habitats and 90% of them live in marine or brackish waters, such that freshwater crustaceans comprise only a small proportion of the group (Kawai and Cumberlidge, 2016). And, as in other freshwater animals, freshwater crustaceans are hyperosmoregulators (Anger, 2003; Anger, 2016). Therefore, a great challenge for freshwater crustaceans is taking up rare ions from the very dilute media of their environment.

Osmotic and ionic regulation have been extensively studied in crustaceans, resulting in many reviews (Potts and Parry, 1964; Mantel and Farmer, 1983; Péqueux, 1995; Harris and Aladin, 1997; Ahearn et al., 1999; Charmantier et al., 2009; Henry et al., 2012; McNamara and Faria, 2012; Larsen et al., 2014; Leone et al., 2017). Also, in addition to ion uptake from the environment, ion reabsorption from urine takes place in excretory organs, such as maxillary or antennal glands, in order to regulate hemolymph osmolality and conserve ions in freshwater environments. The topic of ion reabsorption from urine is covered extensively elsewhere (Harris and Micaleff, 1971; Kamemoto and Tullis, 1972; Peterson and Loizzi, 1974; Riegel, 1977; Mantel and Farmer, 1983; Henry and Wheatly, 1988; Ahearn and Franco, 1990; Sarver et al., 1994; Péqueux, 1995; Wheatly and Gannon, 1995; Vogt, 2002; Khodabandeh et al., 2005a; Khodabandeh et al., 2005b; Khodabandeh et al., 2005c; Freire et al., 2008; Charmantier et al., 2009). Given prior reviews covering various aspects of crustacean osmotic and ionic regulation, the goal here is not to provide a comprehensive review. Rather, the focus here is to present evidence supporting hypotheses of ion uptake, specifically Na^+^ uptake, from very dilute environments (Figure 1) and discuss unresolved issues regarding those mechanisms.

### Ion uptake in gills and extrabranchial organs of crustaceans

In crustaceans, ion uptake from the environment is performed in a wide range of organs, including in pleopods of isopods (Postel et al., 2000), epipodites (= epipods) of branchiopods (Aladin and Potts, 1995), swimming legs of copepods (Johnson et al., 2014; Gerber et al., 2016), and gills of decapod crustaceans (Onken and McNamara, 2002; Cieluch et al., 2005; Henry et al., 2012). In some smaller species, ionic and osmotic regulation are performed on surfaces of the body, such as integumental windows and dorsal organs, as in some syncarids and cladoceran branchiopods (reviewed in Charmantier et al., 2009; Loose et al., 2020). The structures and functions of crustacean iono- osmo-respiratory organs, especially those of decapods, have been reviewed elsewhere (Mantel and Farmer, 1983; Taylor and Taylor, 1992; Péqueux, 1995; Freire et al., 2008; Charmantier et al., 2009; Loose et al., 2020).

Interestingly, a wide array of crustacean iono- and osmoregulatory (and respiratory) organs, such as branchiopod epipodites and decapod gills, are thought to be homologous and developmentally derived from arthropod appendages (legs) (Franch-Marro et al., 2006; Boxshall and Jaume, 2009). The strong association of crustacean osmoregulatory organs with legs is supported by the discovery of ion transporters clustered in “Crusalis organs” of the swimming legs of the copepod *Eurytemora affinis* complex (Johnson et al., 2014; Gerber et al., 2016). Interestingly, development of crustacean epipodites and gills is controlled by orthologs of the master regulator transcription factors *trachealess* (*trh*) and *ventral veinless* (*vvl*) (Mitchell and Crews, 2002; Franch-Marro et al., 2006; Wang et al., 2012), which also control the development of respiratory trachea in insects (Franch-Marro et al., 2006; Chung et al., 2011) and lungs/trachea in vertebrates (Levesque et al., 2007; Zhou et al., 2009).

### Support for model 1 in crustaceans

With respect to the hypotheses described above (Figure 1), available physiological, molecular, and histological data on hyperosmoregulating crustaceans in very dilute environments show predominant support for Model 1 (Figure 1A), or a more complex variant of this model (see e.g., Kirschner, 2004; Bianchini and Wood, 2008; Freire et al., 2008; Charmantier et al., 2009; McNamara and Faria, 2012). Here, we propose two models, either with a single cell or with two associated cells. In both models, the main driving force for ion transport is provided by the apical VHA and the basolateral NKA.

For ion uptake from fresh water, the involvement of VHA has been implicated in several groups of crustaceans (reviews in Charmantier et al., 2009; McNamara and Faria, 2012; Weihrauch and O’Donnell, 2015; Leone et al., 2017). These include crayfish (Zare and Greenaway, 1998), decapod crabs (Putzenlechner et al., 1992; Onken and Putzenlechner, 1995; Riestenpatt et al., 1995; Weihrauch et al., 2001; Genovese et al., 2005; Weihrauch et al., 2005; Tsai and Lin, 2007), palaemonid shrimps (Faleiros et al., 2010; Boudour-Boucheker et al., 2014, 2016; Lucena et al., 2015; McNamara et al., 2015; Faleiros et al., 2017), the amphipod *Gammarus fossarum* (Dayras et al., 2017), calanoid copepods (Lee C. E. et al., 2011; Johnson et al., 2014; Gerber et al., 2016), and neonates of the branchiopod *Daphnia magna* (Bianchini and Wood, 2008). In the copepod *Eurytemora affinis* complex, VHA activity shows both an evolutionary increase in recently derived freshwater populations, relative to saline populations, and an acclimatory increase under freshwater conditions for both saline and freshwater populations (Lee C. E. et al., 2011). This evolutionary increase in activity in freshwater populations suggests its important role in freshwater adaptation.

**FIGURE 2:**
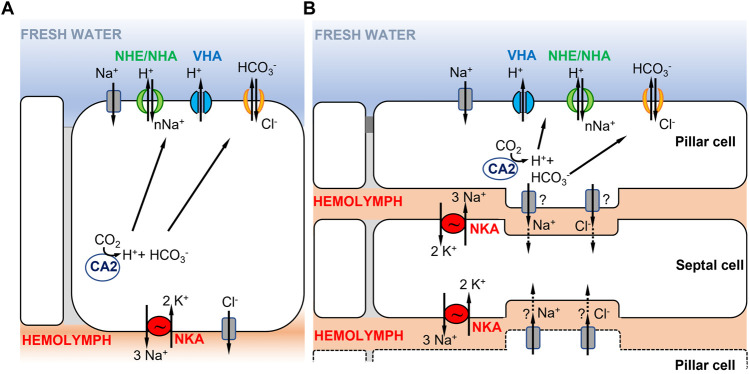
Hypothetical ionocyte models of ion uptake for crustaceans in freshwater habitats. **(A)** A single ionocyte, as found in decapods and particularly in brachyuran crabs (from Towle and Weihrauch, 2001; Charmantier et al., 2009). Under low salinity conditions, the apically localized VHA transports H^+^ out of the cell, driving Na^+^ entry into the cell through an unknown electrogenic Na^+^ transporter. **(B)** Cooperating ionocytes, pillar and septal cells, as found in different species of palaemonid shrimps (adapted from McNamara and Faria, 2012; Boudour-Boucheker et al., 2014). On the apical membrane of pillar cells, VHA drives Na^+^ uptake into the cell as in **(A)**. Na^+^ is then passed to septal cells and then transported to the hemolymph by NKA located on the membrane of septal cells. In both models in **(A,B)**, the type of apical ion transporter responsible for transporting Na^+^ from the environment into the cell is uncertain (see text), as well as the chloride bicarbonate exchangers or channels involved.

The first model for crustaceans, based mostly on studies of decapod crabs, involves a single ionocyte type (Figure 2A). In this model, the electrochemical gradient that drives ion uptake from the environment is generated by the combined actions of VHA and NKA (see previous section) (Figures 1A, 2A) (Charmantier et al., 2009; McNamara and Faria, 2012; Yang et al., 2019; Lee, 2021). This electrochemical gradient drives Na^+^ uptake across the apical membrane (Larsen et al., 2014) by some unknown Na^+^ transporter (i.e., the unknown “Wieczorek Exchanger; ”see below). Na^+^ is then transferred from cytoplasm to hemolymph through the basolateral NKA. Apical entry of Cl^−^ may be mediated by the Na^+^,K^+^,2Cl^−^ cotransporter (Riestenpatt et al., 1996; Towle, 1998; Weihrauch and Towle, 2000; Luquet et al., 2005) or by a Cl^−^/HCO_3_^−^ exchanger (Onken et al., 1991; Genovese et al., 2005) with basolateral transfer to the hemolymph performed by Cl^−^ channels (Bianchini et al., 1988; Siebers et al., 1990; Towle and Smith, 2006). Basolateral K^+^ channels likely enable the recycling of K^+^ used by NKA. The intracellular carbonic anhydrase supplies H^+^ to the proton pump VHA, enabling the apical uptake of Na^+^, and HCO_3_^−^ ions to the apical Cl^−^/HCO_3_^−^ exchanger, in exchange for Cl^−^.

A second model proposed in decapod palaemonid shrimps involves two morphologically distinct ionocytes that cooperate to perform ion uptake from the environment (Figure 2B) (reviewed in McNamara and Faria, 2012; Boudour-Boucheker et al., 2014; McNamara et al., 2015). The two distinct types of ionocytes include pillar cells, which have extensive apical flanges facing the environment, and septal cells, which are in contact with the hemolymph (McNamara and Faria, 2012; Boudour-Boucheker et al., 2014). As shown by their immunostaining, the two main enzymes driving ion transport are located in different cells, with VHA localized on the apical side of the pillar cell flange and NKA on the membranes of septal cells in contact with the hemolymph (Boudour-Boucheker et al., 2014; Lucena et al., 2015; Maraschi et al., 2015; Pinto et al., 2016). On the apical membrane of the pillar cell, VHA generates the electrical gradient that favors Na^+^ uptake from the external medium, through an unknown Na^+^ channel or some other electrogenic Na^+^ transporter (McNamara and Faria, 2012; Boudour-Boucheker et al., 2014). Within the pillar cell, a cytoplasmic carbonic anhydrase (CA) delivers H^+^ to VHA, to enable Na^+^ uptake, and provides HCO_3_^−^ to an apical Cl^−^/HCO_3_^−^ exchanger, permitting Cl^−^ uptake (McNamara and Faria, 2012). Subsequently, Na^+^ and Cl^−^ are either transported directly to the hemolymph space or passed through a junctional complex to the adjacent septal cells. Freire and McNamara (1995) showed that septal and pillar cells of the shrimp *Macrobrachium olfersii* have areas of attachment through desmosomal contact. If passed to the septal cells, Na^+^ would then be transported to the hemolymph by NKA on the membrane of the septal cell, with recycling of K^+^ by hypothetical K^+^ channels. The depletion in Na^+^ content in the septal cell is what drives Na^+^ from the adjacent pillar cell to the septal cell. Cl^−^ exits to the hemolymph through Cl^−^ channels, possibly from either pillar or septal cells (McNamara and Faria, 2012).

For freshwater crustaceans, there is considerable uncertainty regarding various features of the models of ion uptake (Figure 2). A key puzzle in these models regards which ion transporter is apically localized and responsible for transporting Na^+^ into the cell from dilute environments. In crustaceans, three types of transporters have been proposed to mediate Na^+^ entry under freshwater conditions, namely, a Na^+^ channel (NaCh), the Na^+^/H^+^ exchanger (NHE, SCL9A), or the electrogenic Na^+^/H^+^ antiporter (NHA, SLC9B) (Towle and Weihrauch, 2001; Charmantier et al., 2009; McNamara and Faria, 2012; Stern and Lee, 2020). The Na^+^,K^+^,2Cl^−^ cotransporter has been proposed to perform Na^+^ uptake under brackish water conditions (Riestenpatt et al., 1996; Weihrauch and Towle, 2001). As mentioned in the Introduction, the Na^+^ transporter cooperating with VHA was hypothesized to be electrogenic (Wieczorek et al., 1999; Beyenbach and Wieczorek, 2006). As discussed in the following paragraphs, sufficient data are lacking regarding the identities of the secondary transporters that cooperate with VHA to perform the apical uptake of Na^+^ in crustaceans and the topic remains controversial.

The two models (Figure 2) are based mostly on data from decapod crustaceans, which might not be representative of crustaceans as a whole. Within the order Decapoda, multiple independent instances of whole genome duplication have taken place (Lécher et al., 1995; Gutekunst et al., 2018), possibly leading to gene family expansions and the evolution of morphological complexity. Decapods are peculiar among crustaceans in possessing highly derived sets of gills, which are developmentally derived from legs (Franch-Marro et al., 2006). As mechanisms of ion transport have not been studied in most orders of crustaceans, the models based on decapods might not be generalizable to the diversity of mechanisms that could be operating across divergent crustacean taxa.

An apically localized Na^+^ channel (NaCh) was proposed to utilize the voltage gradient generated by VHA to take up Na^+^ by crustaceans under freshwater conditions (McNamara and Faria, 2012). An apical Na^+^ channel was inferred to be involved in ion uptake in fresh water in gill ionocytes of the Chinese mitten crab *Eriocheir sinensis*, based on inhibition of Na^+^ uptake using the pharmacological inhibitor amiloride (Zeiske et al., 1992). However, the use of amiloride cannot clearly distinguish between the effects of Na^+^ channel and NHE (Masereel et al., 2003; Quijada-Rodriguez et al., 2017). Likewise, amiloride and ethylisopropyl-amiloride (EIPA) cannot distinguish between the two models of Na^+^/H^+^ exchange in the crayfish *Procambarus clarkii* (Kirschner, 2002). Thus, the identities of the ion transporters in these studies remain uncertain, as their DNA was not sequenced. Also, a subsequent study in split gill lamellae of the European green crab *Carcinus maenas* showed that most of the amiloride effect might be explained by its inhibition of ion fluxes through the cuticle, rather than *via* Na^+^ channels in the apical membrane (Onken and Riestenpatt, 2002). The involvement of an epithelial Na^+^ channel associated with VHA was inferred in whole-body Na^+^ uptake by neonates of the branchiopod *Daphnia magna*, using the inhibitors phenamil and bafilomycin (Bianchini and Wood, 2008). However, thus far, there is no clear evidence that Na^+^ channels are involved in ion uptake specifically in crustacean osmoregulatory organs, including in crustacean gills or epipodites. Thus, evidence for its role in Na^+^ uptake in fresh water is still limited and inconclusive, requiring much more research.

Despite the presence of Na^+^/H^+^ exchanger (NHE) (SLC9A) in crustaceans, there is no clear evidence for Model 2 (Figure 1B) in freshwater crustaceans. As of yet, no study has conclusively established that NHE functions as an apical ion transporter performing Na^+^ uptake in an electrogenic manner in crustaceans. In the literature on brackishwater decapods, Na^+^ uptake across the apical membrane was claimed to be performed by an electrogenic Na^+^/H^+^ exchanger, where two Na^+^ are exchanged for one H^+^ (Shetlar and Towle, 1989). The stoichiometry of 2Na^+^/1H^+^ exchange was detected using a fluorometric assay based on acridine orange. However, in this study, and other similar studies (Ahearn and Clay, 1989; Ahearn and Franco, 1990), the genetic identity of the Na^+^ transporter studied has been unclear. NHE was inferred to be involved in whole body Na^+^ uptake in adults of the branchiopod *Daphnia magna* (Bianchini and Wood, 2008), but this result was determined using the inhibitor amiloride, which cannot discriminate between Na^+^ channel and NHE (Masereel et al., 2003; Quijada-Rodriguez et al., 2017). Transcripts of the NHE gene have been found to be expressed in the gill tissue of carid shrimp *Macrobrachium amazonicum* (Boudour-Boucheker et al., 2016), the blue crab *Callinectes sapidus,* and the green shore crab *Carcinus maenas* (Towle et al., 1997; Towle and Weihrauch, 2001). However, conclusive links have not been made between expression of *NHE* in crab gills and its stoichiometry of Na^+^ transport.

Rh proteins (RhCM) have been sequenced in crustacean species, notably crabs (Weihrauch et al., 2009), but they differ from vertebrate Rh proteins and have uncertain function. Moreover, the subcellular localization of RhCM in osmoregulatory posterior gills of crabs is not known. Importantly, no evidence is available on the presence of a functional link between ammonia excretion and Na^+^ uptake, as has been described in fish gills (see *Support for Model 2 in teleost fish*, below).

Some empirical results are consistent with the argument that an electroneutral NHE might enable Na^+^ uptake at brackish (low) salinities, and freshwater conditions (Parks et al., 2008; Dymowska et al., 2015). The estuarine carid shrimp *Macrobrachium amazonicum* showed higher gene expression of branchial *NHE* at lower (5 PSU) than at higher (25 PSU) salinity, suggesting a role in ion uptake at lower salinities (Boudour-Boucheker et al., 2016). Similarly, in the penaeid shrimp *Penaeus monodon*, expression of *NHE* was higher in fresh water than in saline media, in conjunction with higher expression of *NKA* and *VHA* (Rahi et al., 2021). However, In the copepod *Eurytemora affinis* complex, some *NHE* paralogs showed increased expression under saline conditions, whereas other paralogs displayed increased gene expression under freshwater conditions (Posavi et al., 2020), suggesting functional differentiation among NHE paralogs. Signatures of positive selection (based on dN/dS ratio) in the *NHE* gene were found in the lineage of the freshwater crab *Eriocheir sinensis*, relative to other decapod crab species, suggesting functional evolution (Wang et al., 2018).

The electrogenic Na^+^/H^+^ antiporter (NHA) (SLC9B) was originally thought to be an exclusively bacterial ion transporter until 2005, when the first DNA sequences of *NHA* were obtained for eukaryotes, including animals (Brett et al., 2005). Phylogenetically, *NHA* sequences were found to form a sister clade with *NHE* (SLC9A) (Brett et al., 2005). Animal NHAs were functionally analyzed only starting in 2007 in insects (Rheault et al., 2007; Day et al., 2008; Xiang et al., 2012). At this point, concrete functional information on NHA in arthropods comes almost exclusively from studies of insects (see next section).

Starting in the late 1980s, kinetic Na^+^ flux analyses suggested the presence of an electrogenic 2Na^+^/1H^+^ exchanger/antiporter in crustaceans that could potentially act as the missing “Wieczorek exchanger” (Shetlar and Towle, 1989; Ahearn et al., 1990; reviews in Ahearn, 1996; Ahearn et al., 2001; Towle and Weihrauch, 2001). Such putatively electrogenic Na^+^/H^+^ antiporters have been found in various decapod crustaceans, such the green crab *Carcinus maenas* (Shetlar and Towle, 1989), the American lobster *Homarus americanus* (Ahearn and Franco, 1990; Ahearn et al., 1994) and the shrimp *Macrobrachium rosenbergii* (Ahearn and Clay, 1989; Kimura et al., 1994; Ahearn et al., 1999). However, the genetic identities of these transporters are unclear, given that amiloride and other pharmacological inhibitors are poor at distinguishing among different sodium transporters (Masereel et al., 2003). Additional studies are needed to determine which Na^+^ transporter was analyzed in these functional assays.

Several evolutionary genomic studies implicate NHA paralogs as important contributors to freshwater adaptation (Posavi et al., 2020; Stern and Lee, 2020; Lee, 2021; Stern et al., 2022). Population genomic studies of the copepod *Eurytemora affinis* species complex have found that gene paralogs of *NHA* exhibit signatures of natural selection between ancestral saline and recently freshwater invading populations (Stern and Lee, 2020; Lee, 2021; Stern et al., 2022). Often the same SNPs (single nucleotide polymorphisms) were under selection across repeated saline to freshwater invasion events, suggesting that the same functional sites are evolving within the ion transporter protein. Experimental evolution studies also revealed *NHA* paralogs as genetic targets of selection during rapid salinity decline in the laboratory (Stern et al., 2022). Of the *NHA* paralogs, the *E. affinis* paralogs *NHA7* and *NHA5* showed evolutionary shifts in gene expression between ancestral saline and recently derived freshwater populations, supporting the importance of NHA function during rapid salinity transitions (Posavi et al., 2020; Lee, 2021). These paralogs also showed acclimatory changes in expression with salinity change (Posavi et al., 2020). Additionally, crustacean species that are prone to crossing salinity boundaries exhibit striking *NHA* family expansions. While most insect genomes appear to possess only two paralogs of *NHA* (Xiang et al., 2012; Chintapalli et al., 2015), the genome of the copepod *E. affinis* complex contains 8 paralogs and that of the amphipod *Hyalella azteca* contains 4 (Poynton et al., 2018; Stern and Lee, 2020; Lee, 2021). The fact that *NHA* paralogs are genetic targets of selection during salinity shifts across multiple studies (Lee, 2021) suggest that they are critically important for freshwater adaptation.

We currently lack sufficient physiological data on NHA paralogs to adequately assess their roles in the models of ion uptake (Figure 1). First of all, functional characterization of ion transport by NHA is needed, such as whether ion transport is electrogenic and which specific ion(s) are being transported. Additionally, we need to establish whether NHA is functionally linked to VHA. We still need to determine whether NHA is apically localized, with VHA, as hypothesized in the models of Na^+^ uptake (Figures 1A, 2). Even for insect model systems, the functions of NHA paralogs are not resolved (Xiang et al., 2012; Chintapalli et al., 2015) (see next section). Detailed research is needed to uncover the functional differences among the different NHA paralogs across a variety of animal models.

The Na^+^,K^+^,2Cl^-^ cotransporter (NKCC) (SLC12A) is thought to play an important role in Na^+^ uptake in “weak hyperregulating” crustaceans in brackish water, but not under freshwater conditions (Towle and Weihrauch, 2001; McNamara and Faria, 2012; Moshtaghi et al., 2018). As of yet, there is no evidence that an apical NKCC performs Na^+^ uptake under freshwater conditions. In brackishwater decapod crustaceans, an apical NKCC has been proposed to perform Na^+^ uptake, in cooperation with K^+^ channels, with this uptake energized by NKA at the basolateral membrane (Riestenpatt et al., 1996; Luquet et al., 2005). For instance, in the green shore crab *Carcinus maenas*, an apical NKCC was proposed to perform Na^+^ uptake in brackish salinity (248 mmol/L NaCl, ∼14.5 PSU) based on voltage clamp and ion flux studies on split gill lamella in an Ussing chamber (Riestenpatt et al., 1996). Likewise, an apical NKCC was hypothesized to perform Na^+^ uptake in the epipodite of the lobster *Homarus americanus* under brackishwater conditions (240 mmol/L NaCl, ∼14 PSU) (Lucu and Towle, 2010). Consistent with apical Na^+^ uptake in brackish water, *NKCC* mRNA expression increased 10-22 fold after transfer from 30 PSU to 2 PSU salinity within 24 h in the posterior gills of the estuarine crab *Neohelice (Chasmagnathus) granulata* (Luquet et al., 2005). In addition, gill NKCC protein synthesis in several *Macrobrachium* species was associated with hyperosmoregulatory capacity at salinities close to 24 PSU, suggesting a role for NKCC in salt uptake at relatively high salinity (Maraschi et al., 2021). Synchronous patterns of gene expression of *NKCC* and other ion transporters (e.g. NHE, CA, NKA) in the posterior gills of the mud crab *Scylla paramamosain* across molt stages suggest that these ion transporters cooperate in function during larval development (Xu et al., 2017). However, more studies are needed to verify whether NKCC is apically localized and which ion transporters are cooperating with NKCC.

### Conclusion and perspectives on models of ion uptake in crustaceans

At this point in time, many fundamental questions regarding mechanisms of ion uptake by crustaceans under freshwater conditions remain unresolved. In particular, the identity of the ion transporter(s) responsible for the apical uptake of Na^+^ from fresh water remains uncertain. It is quite likely that NHA (SLC9B) is of widespread importance across crustaceans, as well as in many other taxa, but many more rigorous functional studies are needed. It is not clear where NHA is localized within ionocytes and which ions different NHA paralogs are transporting, even in model systems, such as *Drosophila melanogaster* (Rheault et al., 2007; Xiang et al., 2012; Chintapalli et al., 2015) (see next section).

Traditional physiological approaches for studying ion transport mechanisms in crustaceans have yielded inconclusive results. Many traditional physiological studies that explore ion transporter functions (often quite elegantly) have not genetically identified the ion transporters under study. Thus, the results on ion transporter function are often decoupled from identities of the actual ion transporters, contributing to uncertainties regarding the roles of Na^+^ channel *vs.* NHE *vs.* NHA. Also, in many cases, it is unclear which ion transporter paralog (gene duplicates with DNA sequence variation) is being studied. Whole genome and transcriptome data are needed in order to identify the full complement of ion transporters that exist within genomes, including all gene duplicates and paralogs belonging to each ion transporter gene family. In many cases, different paralogs of an ion transporter could be performing different functions. Some of the confusion in the literature on the functions of particular ion transporters might arise from differences in function among gene paralogs (Xiang et al., 2012; Lee, 2021). It would also be important to identify the functions of alternative splice variants (isoforms).

In addition, traditional physiological approaches have often applied methods that lack the specificity to distinguish among ion transporters. Models of cellular ion transport have been based largely on biochemical and electrophysiological experiments on perfused gills or split gill lamellae, along with the use of pharmacological inhibitors to evaluate the impact of ion transporters on ion fluxes and transepithelial potential (Siebers et al., 1990; Onken et al., 1991; Zeiske et al., 1992; Onken and Putzenlechner, 1995; Riestenpatt et al., 1996; Postel et al., 2000; Onken and Riestenpatt, 2002). A key problem of these approaches is that pharmacological inhibitors are often insufficiently specific, inhibiting more than one ion transporter. For instance, amiloride cannot distinguish between Na^+^ channels and NHE. Also, many inhibitors block the activity of only a subset of paralogs of a given ion transporter gene family, leading to erroneous conclusions regarding the function of an entire gene family (Masereel et al., 2003). Thus, while use of pharmacological inhibitors could provide useful initial results, such studies should be followed by more rigorous gene specific analyses, such as RNAi, or CRISPR/Cas gene editing approaches. In addition, heterologous gene expression assays, such as the expression of ion transporter genes in *Xenopus* oocytes or yeast, could be used to examine ion transporter function of specific gene paralogs (Piermarini et al., 2009; Piermarini et al., 2010).

Accurately localizing the ion transporters on the apical or basal membranes of ionocytes is a key step toward clarifying their functions, yet this information is lacking for many of the key transporters. The actual localization of critical transporters, largely documented for NKA and to a lesser extent for VHA, is still largely missing for many ion transporters in most crustacean species, such as Na^+^,K^+^,2Cl^−^ cotransporter, Cl^−^/HCO_3_^−^ exchanger, putative Na^+^ channels, NHE, and NHA. Immunolocalization with specific antibodies has proven to serve as a powerful tool. Ideally, the antibodies would be specific to each individual paralog, given that different paralogs of an ion transporter might have different patterns of expression and localization, as well as divergent functions.

Additionally, the ion transporters, especially their paralogs, should ideally be studied in a phylogenetic context. Such an approach would uncover how the ion transporters are evolutionarily related to one another, as well as patterns and rates of evolution. For instance, applying a phylogenetic approach revealed the fact that NHA and NHE form sister clades, rather than belonging to the same gene family (Brett et al., 2005). Phylogenetic placement of the ion transporters allows the detection of the direction of evolutionary changes, such as the sequential order of gene duplications and mutations, identifying which paralogs are ancestral and which are derived. Uncovering the evolutionary history of ion transporters could provide insights into the evolutionary succession of their functional changes. For instance, it would be informative to identify mutational differences among paralogs and determine how the mutational changes affect the evolution of function.

Finally, additional studies are needed on the ontogeny of ion transporter localization and functions during development (Charmantier and Charmantier-Daures, 1994; Charmantier, 1998; Charmantier et al., 1998; Charmantier and Charmantier-Daures, 2001; Charmantier et al., 2001; Cieluch et al., 2004; Khodabandeh et al., 2005a; Khodabandeh et al., 2005c; Cieluch et al., 2007; Charmantier and Anger, 2011; Boudour-Boucheker et al., 2016). Most studies have focused on the adult stages, but adaptation to fresh water might take on different forms over the life cycle of aquatic organisms. Given the changes in body size and anatomy across life stages, physical constraints on ion uptake will change during development. Features of ion transporters that change during development might include the structure of ion transporters, their localization in ionocytes and tissues, their activity and expression, and the stoichiometry of ion transport. Constraints and requirements will vary across life stages, and natural selection in response to salinity stress might act differentially across different life history stages.

## Ion uptake in insects

The Hexapoda, which include the insects, are essentially crustaceans that have colonized terrestrial habitats (Glenner et al., 2006). Phylogenomic analyses date the origin of insects to the Early Ordovician, approximately 479 million years ago (Misof et al., 2014); although, this date precedes earliest evidence of insects in the fossil record of ∼400 million years ago (Engel and Grimaldi, 2004; Garrouste et al., 2012). Both the Hexapoda (i.e., Insecta, Collembola, Protura, Diplura) and the crustacean clades belong the subphylum Pancrustacea, where the Hexapoda are nested within the crustacean clades (Von Reumont et al., 2012; Misof et al., 2014). Thus, insects likely share some basic mechanisms of ion uptake with crustaceans, but with adaptations that reflect its evolutionary history of terrestrialization.

Life on land presents very different challenges from living in water, particularly due to the general lack of aqueous media on land. Physiological mechanisms to address the challenges of water retention include cuticular waterproofing, a tracheal system to reduce respiratory water loss, and the capacity to produce hyperosmotic excreta (Beyenbach and Piermarini, 2008; Bradley et al., 2009). These mechanisms are reviewed in detail elsewhere (Beyenbach and Piermarini, 2008; Bradley et al., 2009). Terrestrial insects no longer take up ions from the surrounding media, but from ingested fluids and food. In terrestrial insects, regulation of fluids and ions occurs mainly in the gut and Malpighian tubules (Beyenbach et al., 2010; Denholm, 2013). Subsequently, the regulation of urine concentration occurs through ion transport in the rectum.

This section focuses mainly on ion uptake mechanisms in aquatic insects, given that freshwater insects take up ions from their surrounding media, unlike terrestrial insects. Of particular relevance here is that larval stages of several insect taxa have secondarily colonized aquatic habitats multiple times independently (Grimaldi and Engel, 2005). For instance, mosquitoes (Diptera: Culicidae), which originated at least 226 million years ago (Reidenbach et al., 2009), have egg and larval stages that occur predominantly in freshwater habitats (95% of mosquito species) (Bradley, 1987; Bradley, 1994).

Mosquito larvae are able to survive in freshwater habitats by reducing drinking to a minimum, producing very dilute urine, and performing active ion uptake into the hemolymph from the external medium (Bradley, 1987, 1994; Bradley et al., 2009). The organs responsible for water elimination and ion conservation and uptake are the midgut, Malpighian tubules, rectum, and anal papillae. In the midgut, nutrients and ions enter the hemolymph from the midgut lumen by active transport, with water following passively. The Malpighian tubules, considered the “insect kidney,” is where primary urine is produced in insects. Here, organic wastes, water, and ions are excreted as a fluid that is isosmotic with the hemolymph. The rectum is particularly important for ion and water uptake, as urine produced by the Malpighian tubules is modified in the rectum, with active transport of ions from the urine into the hemolymph when needed (Smith et al., 2008; Beyenbach, 2016). In the rectum, solutes such as NaCl and KCl that were secreted in the Malpighian tubules can be reabsorbed (Beyenbach, 2016). Under very low salinity, however, the urine cannot be made as dilute as the surrounding waters. In such cases, active ion uptake takes place from the external environment, such as by specialized ionocytes in the anal papillae of freshwater mosquito larvae (Donini and O'Donnell, 2005; Donini et al., 2007; Del Duca et al., 2011; Beyenbach, 2016) or “gills” of mayfly (Bradley, 1994, 2009).

Several studies provide support for VHA as the apical driver for ion uptake in freshwater insects, working in conjunction with basolateral NKA. RT-PCR assays revealed that genes that encode VHA and NKA are expressed in all of the iono- and osmoregulatory tissues of the mosquito *Aedes aegypti* larvae (midgut, Malpighian tubules, rectum, and anal papillae) and adults (stomach, Malpighian tubules, anterior hindgut, and rectum) (Patrick et al., 2006; Durant et al., 2021). Various immunolocalization studies also found that VHA and NKA are expressed in key iono- and osmoregulatory tissues (Patrick et al., 2006; Okech et al., 2008; Smith et al., 2008; White et al., 2013). The critical enzyme carbonic anhydrase (Figure 1) was also found expressed in the rectum and anal papillae of larval mosquito (Smith et al., 2008; Durant et al., 2021).

### Support for models 1 and 2 in mosquito larvae

In terms of sodium transporters, there is relatively good evidence that the Na^+^/H^+^ antiporter (NHA) plays an important role in ion uptake by mosquito larvae from freshwater habitats. Studies on mosquito larvae are notable in being the first to discover NHA in aquatic animals (Rheault et al., 2007). NHA is expressed throughout the ion regulatory organs of mosquito larvae, particularly in the chief sites of ion uptake, the rectum and anal papillae (White et al., 2013; Durant et al., 2021). Gene expression and immunolocalization in mosquito larvae (*Anopheles* and/or *Aedes aegypti*) revealed that NHA is localized in key ion regulatory tissues, such as the gastric caeca, anterior midgut, posterior midgut, proximal Malpighian tubules, rectum, and anal papillae (Rheault et al., 2007; Okech et al., 2008; Xiang et al., 2012; White et al., 2013; Durant et al., 2021). NHA is expressed along the luminal border of the rectum in larvae of the mosquitoes *Anopheles coluzzi* and *A. merus* (White et al., 2013)*,* consistent with a role in ion uptake from the lumen of the rectum. Two paralogs of NHA are expressed in the anal papillae of the larvae of *Aedes aegypti* (Durant et al., 2021). In larvae of the mosquito *Anopheles gambiae*, AgNHA1 was found to be co-localized with VHA on the apical membrane of principal cells in the Malpighian tubules, whereas AgNHA2 was localized apically on the stellate cells in Malpighian tubules (Xiang et al., 2012). Similarly, NHA1 and NHA2 are localized on the apical membrane of epithelial cells in the Malpighian tubules of the fruit fly *D. melanogaster* (Day et al., 2008).

**FIGURE 3:**
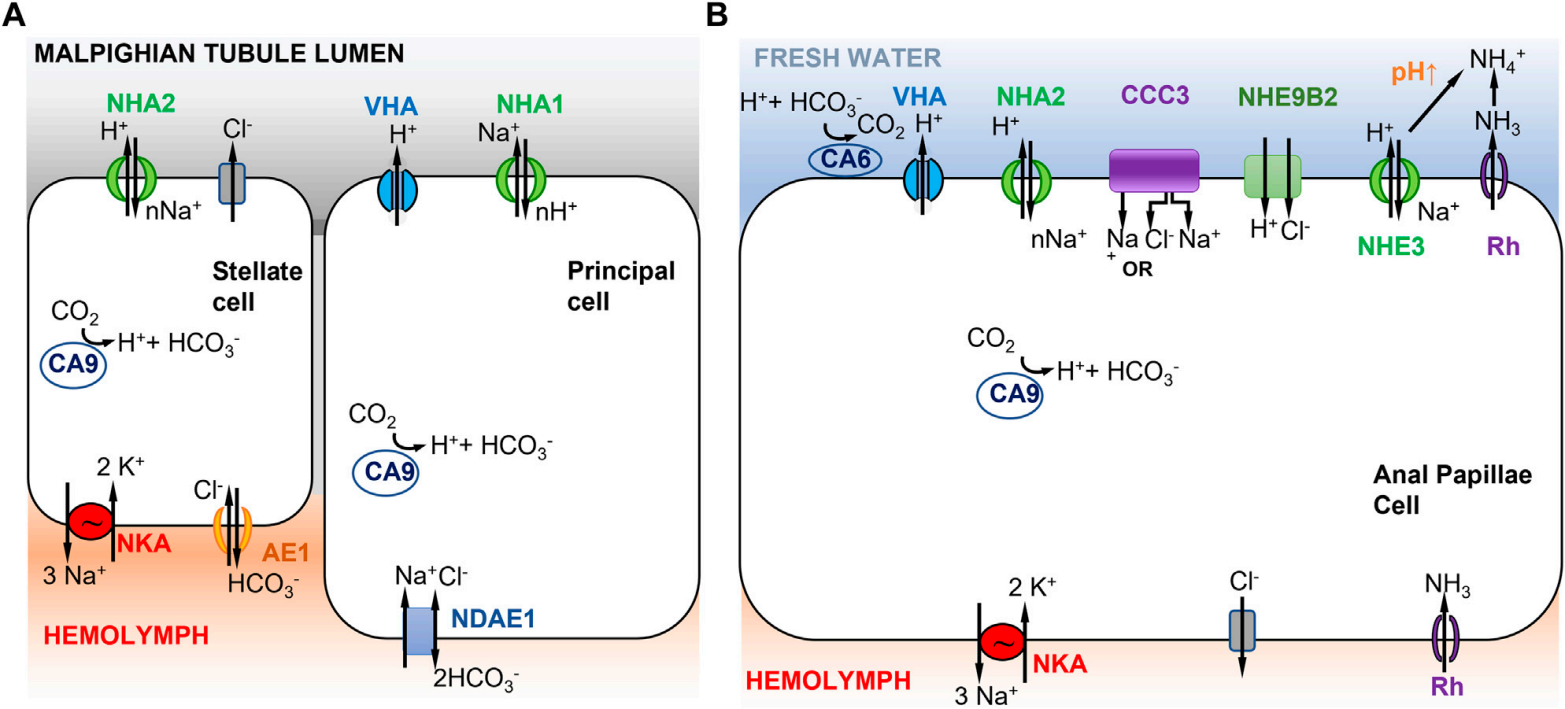
Models of ion uptake by mosquito larvae in freshwater habitats. **(A)** Model of Na^+^ transport in distal Malpighian tubules of mosquito larvae. V-type H^+^ ATPase (VHA) in the apical membrane of principal cells co-localizes with NHA1 (1Na^+^ out to nH^+^ in) and generates a positive voltage that energizes transport events across the apical membrane. The basolateral membrane of the principal cells contains Na^+^ dependent anion exchanger 1 (NDAE1), which translocates 1Cl^-^ and 1Na^+^ into the cells and 2HCO_3_^−^ out. NHA2 in the apical membrane of stellate cells has a proposed stoichiometry of nNa^+^ in and 1H^+^ out. The basolateral membrane of the stellate cell contains Na^+^/K^+^-ATPase (3Na^+^ out and 2K^+^ in) and anion exchanger AE1 (1Cl^-^ in and 1HCO_3_^−^ out). Adapted from Xiang et al. (2012). **(B)** Model of Na^+^ uptake from the external freshwater environment in anal papillae of mosquito larvae. Both Model 1 (Figure 1A) and Model 2 (Figure 1B) are operating here (see text). CCC3 might function as a Na^+^,Cl^−^ cotransporter or Na^+^ transporter. NHE9B2 is homologous to *Drosophila* NHA1, which might function as a H^+^,Cl^−^ cotransporter (Chintapalli et al., 2015) or as a Na^+^/H^+^ antiporter. Adapted from Durant et al. (2021).

Xiang et al. (2012) proposed a model for ion transport in the Malpighian tubules, where mosquito paralogs NHA1 and NHA2 are both electrogenic and differentially localized in two different types of cells, namely, principal and stellate cells (Figure 3A). This model was based on immunolocalization of paralogs of NHA (AgNHA1 and AgNHA2) in the distal Malpighian tubule of larvae of the freshwater mosquito *Anopheles gambiae* and heterologous expression of AgNHA2 in yeast cells. The apical co-localization of AgNHA1 with VHA in the principal cells in the Malpighian tubules suggested that AgNHA1 function is voltage driven (Xiang et al., 2012). In contrast, AgNHA2 was localized on the apical membrane of stellate cells within Malpighian tubules. Yeast cells transformed with AgNHA2 became growth-inhibited when exposed to salts (LiCl, NaCl, and KCl), suggesting greater inward transport of cations (e.g. nNa^+^ in to 1H^+^ out) due to the presence of AgNHA2 (Xiang et al., 2012).

In Xiang et al. (2012)’s model, the principal and stellate cells are interconnected and cooperate to perform ion transport and water secretion (Figure 3A). In the principal cells, VHA pumps H^+^ out into the Malpighian tubule lumen, generating a voltage gradient across the apical membrane. This voltage gradient then drives apical NHA1 to transport nH^+^ into the principal cells and Na^+^ out into the lumen. Then NHA2, apically localized in the stellate cells, drives nNa^+^ (or nK^+^) from the lumen into the cell and H^+^ out to the lumen. Na^+^ is then transported to the hemolymph *via* a basolateral NKA (Xiang et al., 2012). A peculiar feature of this model is that Na^+^ is transported out into the lumen of the Malpighian tubule by the principal cell, and then taken up again by the stellate cell. This mechanism recycles Na^+^ back into the hemolymph to conserve ions from the urine excreted by the Malpighian tubule. Active ion uptake from the urine then takes place in the rectum (Smith et al., 2008). This model is based on immunolocalization of both NHA1 and NHA2, but on an assumed function of NHA1 and indirect inference of NHA2 function (see previous paragraph), such that the validity of this model is not clear. Another type of Na^+^ transporter, the Na^+^ amino acid cotransporters (NATs), might also be performing apical uptake of Na^+^ in the Malpighian tubules. NATs perform amino acid and sodium uptake and have been apically localized in the salivary gland, cardia, gastric caeca, anterior midgut, posterior midgut, and Malpighian tubes of larval *A. gambiae* (Harvey et al., 2009).

In terms of active ion uptake from the external freshwater environment, results from functional ion flux and genome-wide gene expression analysis of anal papillae of mosquito larvae provide some support for Model 1, possibly in combination with Model 2 (Figures 1, 3B) (Del Duca et al., 2011; Durant et al., 2021). The anal papillae of mosquito larvae have important roles in both osmoregulation and ammonia excretion (Wigglesworth, 1933; Bradley, 1987, 1994). Ion flux studies using pharmacological inhibitors had shown that much of Na^+^ uptake in the anal papillae is driven by H^+^ secretion, likely by VHA (Del Duca et al., 2011). Genome-wide gene expression analysis (using RNA-seq) of anal papillae of the larval mosquito *Aedes aegypti* revealed high levels of expression of many subunits of *VHA* in the anal papillae, as well as expression of three paralogs of *carbonic anhydrases* (*CA*) and two paralogs of *NHA* (Durant et al., 2021). Other ion transporters expressed in the anal papillae that could affect sodium transport include several paralogs each of *NKA*, *NHE*, Na^+^-dependent cation-chloride cotransporters (*CCC*), and ammonia transporters (*AMT* and *Rh protein*) (Durant et al., 2021).

Based on these results, a combination of mechanisms could operate in the anal papillae to take up Na^+^ from the freshwater environment (Figure 3B). Apical VHA would pump out H^+^ and drive Na^+^ uptake into the cell (Figure 3B, left) (Del Duca et al., 2011; Durant et al., 2021), consistent with Model 1 (Figure 1A). Na^+^ would then be delivered to the hemolymph *via* NKA. CA would supply H^+^ to VHA. In addition, given the anal papillae’s additional role in ammonia excretion, Na^+^ would also enter the cell during this process (Figure 3B, right), consistent with Model 2 (Figure 1B). Here, ammonia is transported out of the cell by an ammonia transporter (Rh protein). The exported NH_3_ reacts with H^+^ and is converted to NH_4_^+^. This consumption of H^+^ would then drive electroneutral NHE (green) to export H^+^, and consequently import Na^+^. Additionally, the apical aeCCC3 might function as a Na^+^,Cl^−^ cotransporter. However, aeCCC3 might possibly transport only Na^+^, based on results from heterologous expression of a homolog (Kalsi et al., 2019). In either case, apical aeCCC3 would transport Na^+^ inward using the gradient established by the basal NKA, which would keep cytosolic Na^+^ levels very low. At this time, much of this model remains speculative, such that much additional analyses are required to confirm aspects of this model, including localization and co-localization of ion transporter proteins and functional analyses to determine the roles of individual ion transporters.

Functional assays of both NHA1 and NHA2 in the fruit fly *D. melanogaster* appear to tell a different story, based on heterologous expression in *Xenopus* oocytes. Two-electrode voltage clamping was used to measure H^+^, Na^+^, and Cl^−^ transport performed by NHA1 and NHA2 expressed in the oocytes (by measuring pH_i_, *a*Na_i_, and *a*Cl_i_ of oocytes). These assays indicate that NHA1 possibly functions as an electroneutral H^+^,Cl^−^ cotransporter, whereas NHA2 functions as an electroneutral Na^+^/H^+^-exchanger (Chintapalli et al., 2015). These results are not compatible with Xiang et al.’s (2012) two-cell model (previous paragraph), unless *Drosophila* NHA2 is electrogenic. As functional studies of NHA1 and NHA2 are still preliminary, it is still premature to draw any strong conclusions regarding these models of ion uptake. Moreover, given that *Drosophila* is terrestrial, results from this model might be less relevant for ion uptake from freshwater habitats.

NHA functions are likely to be highly divergent among taxa, given recent gene duplications and gene family expansions and lack of sharing of paralogs among different taxa. Unlike *NHE*, which possesses ancient paralogs that had diverged prior to species splits, *NHA* paralogs typically arose after species splits and tend to not be shared (not orthologous) among distinct taxa (Brett et al., 2005; Rheault et al., 2007; Stern and Lee 2020; Stern et al., 2022). In particular, the NHA1 and NHA2 paralogs of the insect order Diptera (e.g., flies, mosquitoes) are not orthologous with “NHA1” and “NHA2” outside of dipterans, such that functional studies of these paralogs in mosquitoes and flies might not have broad relevance outside this particular insect order. Additionally, several arthropod species outside of insects possess more than two paralogs that are unique to a lineage, such as the 8 distinct and unique *NHA* paralogs in the genome of the copepod *E. affinis* complex (Stern and Lee 2020) and 4 unique *NHA* paralogs in the amphipod *Hyalella azteca* (Poynton et al., 2018). These distinct paralogs could potentially all differ in function from one another, lacking orthologs in other taxa.

Models of NHA function could become even more complex, given that there are cases where this class of ion transporters (i.e., the CPA2 superfamily) could function facultatively as ion channels (Fujisawa et al., 2007). In general, the strict dichotomy between ion channels and ion transporters often does not hold, as ion transporters could act as ion channels under certain conditions (DeFelice and Goswami, 2007; Barneaud-Rocca et al., 2011). For example, ancillary proteins modulate the cation flux activity of the cation/H^+^ antiporters (CPAs) of bacteria, such that the transporters act as cation channels in the absence of their ancillary proteins (Fujisawa et al., 2007). Such results indicate the need to explore the conditions under which ion transport stoichiometry might become altered.

### Conclusion on ion uptake in insects, focusing on NHA

Our understanding of ion transport mechanisms is still nascent, even in insects that serve as model systems. We require more comprehensive models of ion transport for all iono- and osmoregulatory organs and tissues of insects, including the alimentary canal, Malpighian tubules, and anal papillae. In particular, much more functional analyses are needed to elucidate the roles of specialized ionocytes of mosquito larvae and the ion transporters embedded within them. Of particular interest are the ionocytes of the anal papillae and rectum and the principal and stellate cells of Malpighian tubules. Also, the functions of different cell types and tissues could vary at different times and in different contexts.

While NHA has been studied most extensively in insects among animals, the functions of different insect NHA paralogs are still not resolved and require additional functional studies. Far more studies are needed to determine whether mosquito NHA1 and NHA2 are electrogenic or electroneutral, which ions are transported, and how their functions vary in different tissues and among taxa. In addition, it would be important to explore conditions under which functions of this ion transporter might become altered, given that this family of ion transporters (as well as other transporter families) have been found to function alternately as channels in bacteria (Fujisawa et al., 2007).

Additionally, it would be critical to extend the functional study of NHA beyond insects, given that NHA appears to be important for ion uptake at very low salinity. Aquatic insects are evolutionary constrained by being secondarily aquatic (from a terrestrial ancestor), such that they likely have many peculiarities that would not apply generally to other systems. It would be particularly interesting to explore the functions of the diverse NHA paralogs in crustaceans and other arthropod lineages. Exploring the roles of NHA during freshwater adaptation in diverse lineages would likely yield many insights into the evolution and functions of this intriguing ion transporter gene family.

## Ion uptake in teleost fish

Teleost fish comprise around 25000 species, constituting the most evolutionary diverse group of vertebrates. At least three rounds of whole genome duplication events (Taylor et al., 2001, 2003; Jaillon et al., 2004), as well as increased complexity of regulatory networks controlling gene expression, have greatly influenced mechanisms of fish physiology, including freshwater adaptation (Meyer and Van de Peer, 2005). The third whole genome duplication event, called the “fish-specific genome duplication” (FSGD or 3R), occurred around 350 million years ago (mya) in teleost fish but not in terrestrial vertebrates (for more details on FSGD, see Meyer and Van de Peer, 2005). The salmonid genome duplication event that occurred afterward, about 50–100 mya, has been hypothesized to provide the genetic material for the evolution of anadromy, enabling salmonids to migrate between freshwater and marine habitats (Allendorf and Thorgaard, 1984). Regarding freshwater adaptation, these genome duplication events are thought to have contributed to the diversification of adaptive strategies among teleost species, or even among populations within a species, in order to cope with different salinity regimes (Dalziel et al., 2014).

Several excellent reviews on osmoregulation are available for teleost fish (Evans et al., 2005; Hwang and Lee, 2007; Hwang et al., 2011; Hiroi and McCormick, 2012; Takei et al., 2014; Zimmer and Perry, 2022), including those with a focus on freshwater environments (Kirschner, 2004; Evans 2011; Hwang, 2011; Dymowska et al., 2012; Zimmer et al., 2017). However, only a few species have been thoroughly investigated, among them zebrafish *Danio rerio*, Mozambique tilapia *Oreochromis mossambicus*, killifish *Fundulus heteroclitus*, medaka *Oryzias latipes* (Hsu et al., 2014), and rainbow trout *Oncorhynchus mykiss* (Dymowska et al., 2012). For other ecologically important species, further investigations are required to construct models of ion uptake and determine the role of each ionocyte subtype according to habitat type.

The functions of organs involved in ionic and osmotic regulation in teleost fish are relatively well characterized (see references above). Under freshwater conditions, fish undergo passive osmotic influx of water and diffusive loss of ions (mainly Na^+^ and Cl^−^). Maintaining blood osmolality at relatively constant levels of around 260–380 mOsm/Kg, depending on the species, is accomplished in juvenile and adult fish by having (1) low integument and gill permeability to ions, (2) low water permeability of distal renal tubules, to avoid excess entry of water through the renal route, (3) reduced or even absent drinking rate and (4) development of elaborate mechanisms of ion transport through, essentially, the gills and the kidney. At the kidney level, these strategies lead to the production of large amounts of dilute urine (Hickman and Trump, 1969).

The gills of adults and skin of young larvae are the main sites of ion uptake in teleost fish. At the early life history stages (i.e., embryos and larvae) the tegument, including the yolk sac, is essential for transepithelial ion transport through integumental ionocytes (Hiroi et al., 2005; Varsamos et al., 2005; Inokuchi et al., 2022). In contrast, for juvenile and adult fish, the main osmoregulatory organs involved in ion uptake from fresh water are the gills (Varsamos et al., 2005; Evans, 2011). The kidney has been much less studied, but is also an important organ involved in the re-uptake of ions from the renal lumen (Hickman and Trump, 1969; Nebel et al., 2005; Madsen et al., 2020). The gut of freshwater-acclimated or freshwater fish does not play an important role in hyperosmoregulation. As water uptake is not required in freshwater habitats, the drinking rate is generally 10–50 times lower in freshwater than in marine fish (Fuentes and Eddy, 1997; Varsamos et al., 2004; Wilson, 2011). The diet can provide a source of ions, notably in species that have low capacities of branchial (gill) Cl^−^ uptake (i.e., killifish, eel, bluegill) or species living in ion-poor environments (reviewed in Wilson, 2011).

Ion uptake within gills of adult teleost fish and skin of young larvae is achieved mainly through active transport occurring in specialized ion-transporting cells, namely ionocytes. Ionocytes in the gills of fish are localized in filaments (interlamellar surfaces) and in lamellar surfaces (Evans et al., 2005). Their number, size, and specific position on the gill epithelium become altered according to salinity and other environmental factors, such as temperature (Mitrovic and Perry, 2009; Masroor et al., 2018), pH (Goss et al., 1998), oxygen levels (Sollid and Nilsson, 2006), and also according to species (Evans et al., 2005).

**FIGURE 4:**
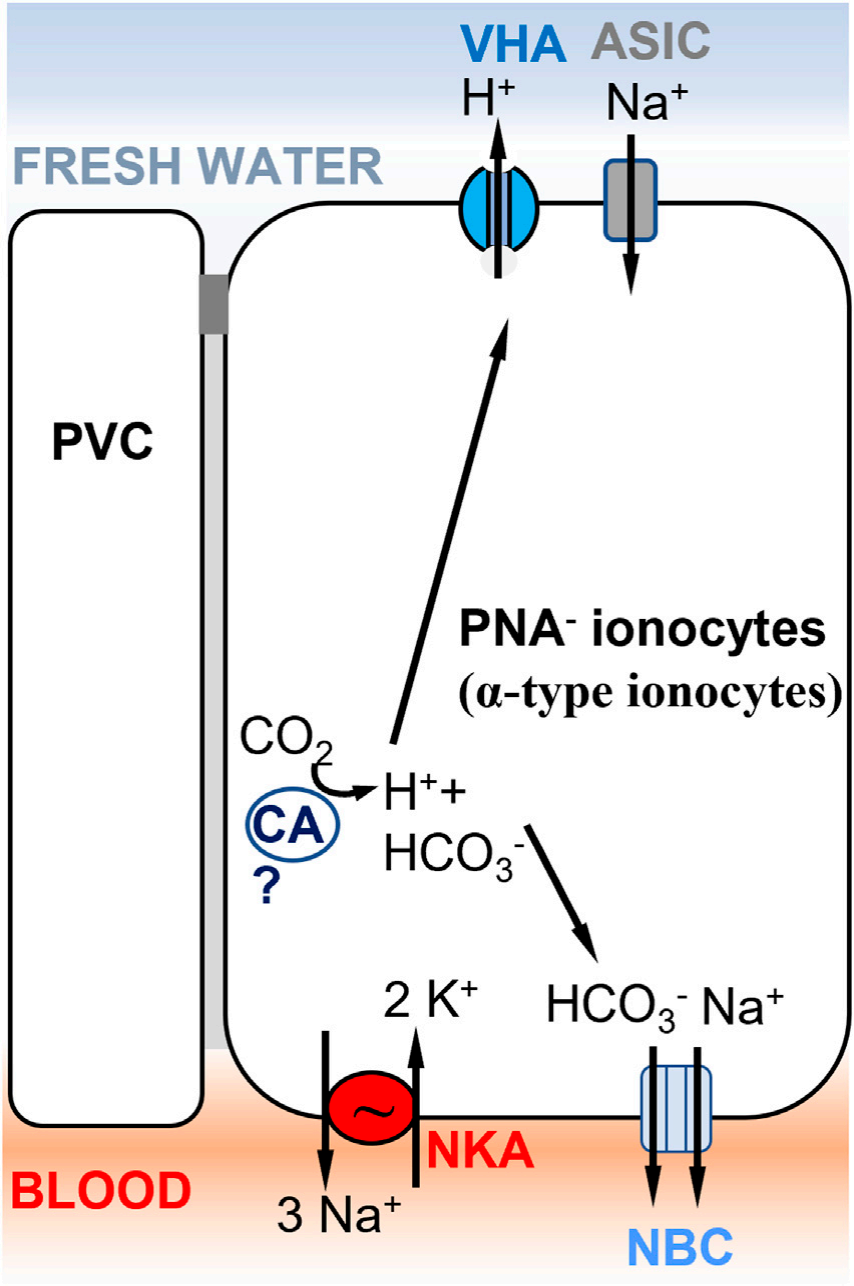
Ionocyte model supporting Model 1 (Figure 1A) in rainbow trout gills, involving ASIC as the Na^+^ transporter. A PNA^−^ ionocyte model (Zimmer et al., 2017) with apical VHA (Lin et al., 1994) electrogenically coupled to a Na^+^ channel, which has been identified as ASIC-4 by Dymowska et al. (2014). PNA^−^ ionocytes do not express NHE2/NHE3 and do not express Rh transporters. A basolateral electrogenic NBC transporter (3? HCO_3_^−^: 1 Na^+^) has been shown to be present by Parks et al. (2007). The presence of a cytoplasmic carbonic anhydrase (CA) is hypothesized to support acid secretion in trout gills, but evidence of the specific CA paralog expressed in this cell type is lacking (Georgalis et al., 2006). PVC= pavement cells.

**FIGURE 5:**
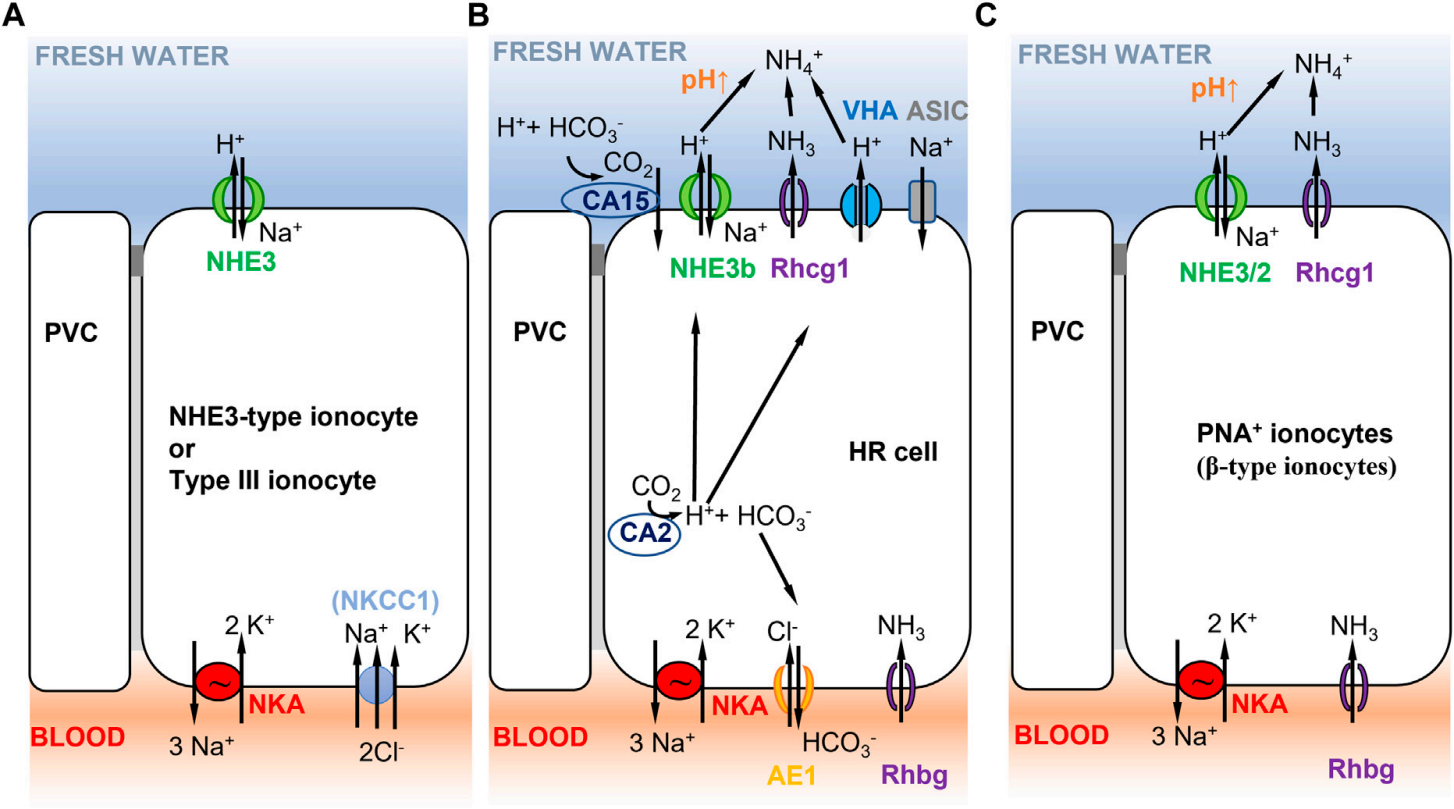
Selected ionocyte models consistent with Model 2 (Figure 1B) in different teleost fish species. **(A)** Tilapia type III ionocyte, and Japanese and European sea bass *D. labrax* NHE3-type cell. Type III ionocyte in tilapia involves apical NHE3 and basolateral NKA, and NKCC1 in fish that have been recently transferred to freshwater (Hiroi et al., 2005, 2008; Inokuchi et al., 2008). NHE-3 cells show similar characteristics and are present in gill lamellae of Japanese (Inokuchi et al., 2017) and European sea bass (Blondeau-Bidet et al., 2019). **(B)** zebrafish HR cell. Na^+^/NH_4_^+^ exchange complex in zebrafish HR cells (Hwang et al., 2011) with basolateral NKA (low expression, mainly *ATP1a1a.5* and *ATP1b1b* paralogous genes, Liao et al., 2009), AE1 (*SLC4a1b*, Lee Y. C. et al., 2011), and Rhbg. HR cells express apical NHE3b, VHA (Yan et al., 2007), Rhcg1, and the acid-sensitive ion channel ASIC (ASIC4.2 in adults, Dymowska et al., 2015; ASIC4b in larvae, Zimmer et al., 2018). Cytoplasmic CA2 contributes to H^+^ production that is apically excreted *via* NHE3b or VHA. Extracellular CA15 (or CA4-like) contributes to lower apical H^+^, resulting in increased pH that favors NHE3b activity. No NBC1 cotransporter has been reported in this cell type (Lee Y. C. et al., 2011). **(C)** Rainbow trout PNA^+^ ionocyte. PNA^+^ ionocyte model with apical NHE3/2 (*slc9a2*, *slc9a3*; Ivanis et al., 2008) and Rhcg1 coupled to basolateral NKA.

Concerted efforts have been made in the past decade to identify different ionocyte subtypes involved in ion uptake. Branchial (gill) NaCl uptake mechanisms have been shown to differ significantly between species (Hwang, 2011). Depending on the fish species, different nomenclatures are used to characterize ionocyte subtypes, such as peanut lectin agglutinin-positive (PNA^+^, Figure 5C) or negative (PNA^−^, Figure 4) ionocytes in rainbow trout (Galvez et al., 2002) *versus* VHA-rich cells (HR-cells, Figure 5B), NKA-rich cells (NaR; not shown), and Na^+^,Cl^−^-cotransporter-expressing cells (NCC-type ionocytes; Figure 1C) in zebrafish skin and gills (Hwang and Lee, 2007). This diversity of ionocyte subtypes between species might reflect different ion uptake mechanisms in different environments. Fish evolving in divergent types of freshwater habitats (differing in pH, oxygen, ionic strength, etc.) and having different life history strategies, will have diversified ion uptake mechanisms. Moreover, ion transporters in these ionocytes are also involved in physiological functions other than osmoregulation, such as acid-base regulation and nitrogen excretion. Thus, the regulation and expression of a set of cooperating ion transporters depend not only on salinity, but also on other environmental factors, such that the transporters must accommodate and compromise their functions across their diverse roles (Evans et al., 2005).

Diverse ion uptake mechanisms have been reported in freshwater-acclimated euryhaline fish or freshwater species (notably zebrafish) (Galvez et al., 2002; Hwang and Lee, 2007; Hiroi et al., 2008), supporting Models 1, 2 and 3 described above (Figure 1). Ionocyte subtypes all express basolateral NKA, which is a crucial enzyme allowing ion transport to the blood, as previously shown in crustacean and insect models (Figures 2, 3). Some of the ionocytes also express VHA (apically or basally located, depending on the models), which can be functionally linked to Na^+^ uptake (see Model 1, Figure 1A). Whether the expression of different cooperating ion transporters and channels are localized in basolateral or apical membranes determines their role in Na^+^ uptake, Cl^−^ uptake, or acid-base regulation (through the transport of acid, H^+^, and base, HCO_3_^−^, equivalents).

The study of freshwater adaptation and plasticity is now being greatly facilitated by the identification of new genes (including gene paralogs) involved in mechanisms of freshwater adaptation and acclimation. The availability of several new fish genomes in the last 20 years (Aparicio et al., 2002; Jaillon et al., 2004; Tine et al., 2014) (available on Ensembl, http://www.ensembl.org) has improved our capacity to identify relevant candidate genes, including different gene paralogs within the same gene family. With these genes, we can explore their functions involved in adaptation or acclimation using various approaches, such as quantifying their expression levels in ecologically important populations or species, performing gene knockdown studies, or examining genetic/genomic signatures of natural selection. Some of the recent studies that use mechanistic approaches are included in the examples below.

The following sections mainly address Na^+^ uptake mechanisms in fish gills or skin (of embryos and larvae), which have been the focus of most research at the cellular and molecular levels in fish. We include findings related to recently discovered ion transporter paralogs that could contribute to freshwater adaptation and acclimation. Based on available data, we describe models of ion uptake reported in teleost fish, in comparison with crustacean and insect models.

### Diverse apical Na^+^ uptake pathways in teleost fish gills

Studies show that within species, different Na^+^ and Cl^−^ uptake pathways exist that involve different cell types. For instance, in zebrafish, five different ionocyte subtypes have been identified that express different sets of cooperating ion transporters (Guh and Hwang, 2017). Within one specific cell type (e.g., the HR cell-type in zebrafish), different Na^+^ uptake pathways can be present, such as an apical Na^+^ channel coupled to VHA (Model 1) and/or an apical NHE3 exchanger coupled to ammonium transport *via* apical Rhcg1 (Model 2). Various Na^+^ uptake pathways coexist within a species, leading to the redundancy of Na^+^ uptake systems. Each Na^+^ uptake pathway seems to vary in its optimal environmental performance, leading to different expression patterns of different transporters depending on the environmental conditions (pH, Na^+^ levels, etc.) (Dymowska et al., 2015).

Early studies have reported that the uptake of Na^+^ *vs* *.* Cl^−^ are independent of each other. Their uptake occurs most likely through ion exchangers involving H^+^/NH_4_^+^ exchanged with Na^+^ and HCO_3_^−^/OH^−^ exchanged with Cl^−^, such that electroneutrality is maintained across the gill epithelium (Smith, 1930; Krogh, 1937). This mechanism of Na^+^ and Cl^−^ uptake has been verified in later investigations (García Romeu and Maetz, 1964; Avella and Bornancin, 1989) and also more recently (Weihrauch et al., 2009; Wright and Wood, 2009; Dymowska et al., 2014). Recent studies also consider elevated K^+^ as a counter ion for Na^+^ uptake in adult zebrafish under acid exposure (Clifford et al., 2022). The uptake of Na^+^ in exchange for protons provides some support for Models 1 and 2 (Figures 1A,B, see next section). In some species, such as the eel *Anguilla anguilla* and bluegill *Lepomis macrochirus*, the gill epithelium is involved in active absorption of Na^+^ with the lack of or minimal Cl^−^ uptake (Scott et al., 2005; Tomasso and Grosell, 2005). This pattern of uptake is consistent with the hypothesis of independent Na^+^ and Cl^−^ uptake mechanisms in fish gills and precludes the need for the ionocyte type in Model 3 for coupled Na^+^ and Cl^−^ uptake (Figure 1C) for the aforementioned species.

More recent findings include the presence of Na^+^, Cl^−^ cotransporters (NCC) in gills of numerous fish species (reviewed in Hiroi and McCormick, 2012), such as European sea-bass (*Dicentrarchus labrax*) (Lorin-Nebel et al., 2006), Japanese sea-bass (*Lateolabrax japonicus*) (Inokuchi et al., 2017), Mozambique tilapia (*Oreochromis mossambicus*) (Hiroi et al., 2008; Inokuchi et al., 2008), Indian medaka (*Oryzias dancena)* (Kang et al., 2010), killifish (*Fundulus heteroclitus)* (Katoh et al., 2008), sailfin molly (*Poecilia latipinna)* (Yang et al., 2011), and zebrafish (Wang et al., 2009). In some of the species mentioned above, a non-specific heterologous antibody (called T4) that recognizes NKCC1, NKCC2 and NCC was used in *in situ* localization studies. The apical staining detected in gill ionocytes of these species was later shown to be NCC2 (reviewed in Hiroi and McCormick, 2012). Subsequent studies have used specific NCC antibodies and confirmed its apical localization (Horng et al., 2009; Hsu et al., 2014). These findings indicate that in some specific ionocyte subtypes of several species, Na^+^ and Cl^−^ transport might be partially linked. Interestingly, salmonid species, and in general migratory species analyzed thus far, do not appear to express the branchial NCC (reviewed in Hiroi and McCormick., 2012).

Thus, Na^+^ uptake models have become increasingly complex and diversified with the discovery and molecular characterization of new ion transporters and their paralogs. Accumulating evidence suggests the presence of three different Na^+^ uptake mechanisms at the apical membrane of branchial ionocytes across different teleost fish taxa, consistent with all three models depicted in Figure 1, namely, (1) Na^+^ uptake coupled to VHA (Model 1), (2) Na^+^/H^+^ exchange (through electroneutral NHE2 or NHE3) linked to apical ammonium excretion through Rhcg (Model 2), (3) Na^+^,Cl^−^ cotransport (NCC-like) coupled to basolateral NKA (Model 3) (Hwang and Lee, 2007; Evans and Claiborne, 2009; Wright and Wood, 2009; Evans, 2011; Dymowska et al., 2014).

### Apical Na^+^ uptake, a long debate regarding models 1 versus 2

Pathways of apical Na^+^ uptake, whether they involve Na^+^ channels (Figure 1A, Model 1) or NHE2/NHE3 (Figure 1B, Model 2), have been debated for numerous years (for details, see Evans, 2011; Dymowska et al., 2012). Na^+^/H^+^ exchange through NHE had been suggested as early as the 1970’s (Kerstetter et al., 1970); however, Na^+^ concentration of freshwater environments is considered too low for NHE to function properly, notably at low pH (Parks et al., 2008). Thermodynamic constraints on the function of NHEs exist at low Na^+^ concentrations (Na^+^ < 0.1 mmol L^−1^) and low pH (pH < 5) levels (Parks et al., 2008), despite the contribution of basolateral NKA to lower intracellular Na^+^ and drive apical Na^+^ uptake (Kirschner, 2004; Parks et al., 2008). For this reason, an alternative Na^+^ uptake mechanism involving an epithelial Na^+^ channel coupled to apical VHA, rather than NHE, has been considered far more plausible (Parks et al., 2008). Although, NHE could function to import Na^+^ under low Na^+^ conditions in the context of a Na^+^/NH_4_^+^ exchange complex (such as with Rh in Model 2, see below).

#### Support for model 1 in teleost fish

Among diverse functions reported for VHA in aquatic organisms (Tresguerres, 2016), H^+^ excretion coupled to Na^+^ uptake is crucial in some freshwater-acclimated species or strictly freshwater fish, like zebrafish (Goss et al., 1998; Perry et al., 2003; Hwang and Lee, 2007). The role of VHA appears crucial, given the steep concentration gradient between the water and the blood (estimated at around 1800 fold for Na^+^ in Hwang, 2011). Branchial VHA is considered to be mainly apically localized in ionocytes (PNA^−^ ionocytes, see Figure 4) of rainbow trout, mudskipper, and zebrafish HR cells (Figure 5B) (Lin et al., 1994; Wilson et al., 2000b; Lin et al., 2006). In some species, however, VHA has not been localized, because no commercial antibodies are available. VHA is highly expressed in rainbow trout PNA^−^ ionocytes (Figure 4) and zebrafish HR cells (Figure 5B). Both cell types are involved in Na^+^ uptake and acid excretion (Reid et al., 2003; Lin et al., 2006). Several studies have also identified in these cells the importance of carbonic anhydrase (CA) paralogs, which perform the hydration of CO_2_ to H^+^ and HCO_3_^−^ (as in *CA2-like a* and *CA15a* in zebrafish HR-cells, Lin et al., 2008) (Figure 5B). H^+^ is then exported through VHA or NHE, whereas HCO_3_^−^ is exchanged with Cl^−^ through the basolateral anion exchanger (AE1) (Figure 5B). Contrary to zebrafish HR cells (Figure 5B), VHA of trout (in PNA^−^ ionocytes) does not colocalize with NHE2/3 (Figure 4), which is present in another cell type (the PNA^+^ ionocyte) (Figure 5C) (Ivanis et al., 2008).

In teleost fish, as epithelial Na^+^ channels (ENaCs) have not been discovered, they are unlikely to serve as the Na^+^ transporter that cooperates with VHA to transport Na^+^ into ionocytes. However, some inconclusive data suggest its presence in teleost fish, though not well substantiated. For instance, in trout PNA^−^ cells, the pharmacological inhibitors phenamil (considered a specific Na^+^ channel blocker (Garty and Palmer, 1997)) and bafilomycin (a VHA blocker) reduced acid-stimulated Na^+^ influx (Reid et al., 2003). Other studies showed phenamil-sensitive Na^+^ transport in teleost species (Bury and Wood, 1999; Grosell and Wood, 2002; Parks et al., 2007), but without any molecular evidence of the presence of ENaCs. A single study using immunolocalization found the apical presence of ENaC in ionocytes and PVCs of trout, with colocalization with VHA (Wilson et al., 2000a). However, this study used a heterologous antibody that was not specifically raised against trout ENaC. As molecular evidence is lacking that ENaC is the phenamil-sensitive Na^+^ channel and, more generally, ENaC is absent in published teleost genomes (Hwang and Lee, 2007), ENaCs are no longer considered to be the Na^+^ transporter that cooperates with VHA in teleost ionocytes.

Rather than ENaC, the acid-sensing ion channel (ASIC) could be the missing apical Na^+^ channels in freshwater-type ionocytes of teleost gills (Montalbano et al., 2021) (Figure 4). Investigations combining the use of pharmacological inhibitors, immunocytochemistry, and mRNA expression identified ASIC channels in trout and zebrafish gills (Dymowska et al., 2014; Dymowska et al., 2015). ASIC is a member of the H^+^-gated Na^+^ channel subfamily, belonging to the amiloride-sensitive ENaC/DEG (Degenerin)/ASIC superfamily of ion channels (Waldmann et al., 1997). Interestingly, ASIC4 is expressed in apical cell membranes of trout NKA-rich ionocytes (called PNA^−^ ionocytes) (Dymowska et al., 2014) (Figure 4) and zebrafish HR cells (Figure 5B) (Dymowska et al., 2015). In those cell types, VHA is believed to export H^+^, leading to acidification and opening of ASICs for enhanced Na^+^ uptake. In juvenile rainbow trout, ASIC-specific pharmacological inhibitors (Chen et al., 2010), diminazene and DAPI, decreased Na^+^ uptake rates in a dose-dependent manner (Dymowska et al., 2014). Some evidence in adult zebrafish supports the role of ASICs in Na^+^ uptake, dependent on external Na^+^ concentrations (Dymowska et al., 2015).

More recently, knockdown studies of *ASIC4b* in zebrafish embryos and the use of DAPI have, however, shown no effect on whole-animal Na^+^ uptake (Zimmer et al., 2018). *ASIC4b* knockdown has led to significant increases in *NHEb* and *NCC* mRNA expression, suggesting that Na^+^ uptake was rescued through other pathways. Interestingly, *ASIC4b* knockdown stimulated Na^+^ uptake in acidic water, suggesting other unidentified Na^+^ uptake mechanisms at low pH (Clifford et al., 2022).

ASICs have been analyzed in only a few species (i.e., rainbow trout and zebrafish) (Zimmer et al., 2018; Clifford et al., 2022). Gene expression levels of *ASIC4* in European sea-bass exposed to fresh water were undetectable in gills, making ASIC4 an unlikely candidate for branchial Na^+^ uptake in this marine species (Blondeau-Bidet et al., 2019). Additional studies on ASIC ion channels in freshwater species, but also in marine and euryhaline species (as a comparison), are required to fully support the role of ASIC as the Na^+^ transporter in Model 1 (Figures 1A, 4).

#### Support for coexistence of models 1 and 2 in ionocytes of some teleost fish

Two Na^+^ uptake mechanisms coexist in the zebrafish HR cell (Figure 5B), supporting both Models 1 and 2 (Figures 1A,B). HR cells express apical VHA coupled to ASIC-4 (see previous section) and also express an apical NHE3b exchanger (see next section for more details). NHE is considered electroneutral in mammalian vertebrates, as well as in fish (Demaurex et al., 1995; Ito et al., 2014). Numerous studies showed that both Na^+^ uptake mechanisms are present in zebrafish HR cells, including those using pharmacological inhibitors (Fenwick et al., 1999; Ito et al., 2014), zebrafish *NHE3b* expression in *Xenopus* oocytes (Ito et al., 2014), and *VHA* knockdown approaches (Horng et al., 2007). The paucity of NKA in HR-cells in zebrafish embryos emphasizes the importance of VHA in providing the energy for Na^+^ uptake (Lin et al., 2006; Esaki et al., 2007; Horng et al., 2007).

Interestingly, the predominance of individual Na^+^ uptake pathways (either NHE3 or VHA coupled to a Na^+^ transporter) seems to depend on environmental conditions. In low-Na^+^ freshwater, for example, mRNA of *NHE3* is upregulated, whereas *VHA* is downregulated in zebrafish gills. An opposite trend in the expression pattern is observed in acidic fresh water (Yan et al., 2007). It thus seems that the partitioning of each Na^+^ uptake pathway depends on the environmental condition in which zebrafish reside, with Model 1 (Figure 1A) being favored in acidic environments and Model 2 (Figure 1B) being favored in low-Na^+^ environments.

#### Support for model 2 in teleost fish

Several lines of evidence support the presence of a Na^+^/NH_4_^+^exchange complex (shown in Model 2, Figure 1B), partially supporting the model initially proposed by Krogh (1937). This model combines Na^+^ uptake, acid secretion, and ammonia excretion. The model was developed for gills of zebrafish (Figure 5B), mangrove killifish (*Kryptolebias marmoratus*) (Wright and Wood, 2009), and medaka larvae (Wu et al., 2010) based on the discovery that Rhesus (Rh) glycoproteins are involved in ammonia transport in aquatic animals (Weihrauch et al., 2004; Hung et al., 2007; Nakada et al., 2007; Nawata et al., 2007). There is also evidence for this model from other freshwater teleost or euryhaline fish transferred to low salinity (Tsui et al., 2009; Weihrauch et al., 2009; Wood and Nawata, 2011; Hsu et al., 2014).

In this model (Figures 5B,C), apical NHE (2 or 3) (and VHA, if present) transports H^+^ out of the cell, resulting in acidification directly outside the apical membrane. This acid-trapping mechanism facilitates NH_3_ (base) excretion *via* an ammonia transporter (Rh protein) (basolateral transport from blood to the cell *via* Rhbg, followed by apical transport from the cell to the external water *via* Rhcg1 or 2). Extracellular NH_3_ then reacts with H^+^ to form NH_4_^+^ in the mucus covering the apical cell membrane. An extracellular carbonic anhydrase (*zCA15a* in zebrafish HR cells) likely contributes to H^+^ production *via* CO_2_ hydration (Lin et al., 2008; Weihrauch et al., 2009). Production of NH_4_^+^, from excreted NH_3_ (*via* Rhcg1 or 2) and H^+^ (*via* VHA or NHE), locally increases the pH, favoring NHE activity to take up Na^+^ and excrete H^+^. Intracellular Na^+^ then enters the blood stream *via* basolateral NKA and/or a Na^+^,HCO_3_^−^ (NBC1) cotransporter.

Support for this model is substantial, as various Rh genes have been sequenced in gills of numerous fish species (Huang and Peng, 2005; Nakada et al., 2007; Nawata and Wood, 2008) and have been localized to branchial ionocytes and PVC in apical (Rhcg1+2) and basolateral (Rhbg) membranes. For instance, morpholino gene knockdown and immunolocalization in zebrafish showed the importance of apical Rhcg1 in ammonia excretion in HR cells (Figure 5B) (Nakada et al., 2007; Shih et al., 2008). Localization of Rhcg1 and Rgbg in the same cell expressing NHE was also reported in ionocytes of medaka fish, with increased expression levels upon low Na^+^ acclimation (Wu et al., 2010). *Rhcg2* mRNA upregulation has also been reported in cultured branchial cells of rainbow trout in low-Na^+^ media (Tsui et al., 2009), but seems to be specific to pavement cells (Zimmer et al., 2017). Zimmer et al. (2017) also showed colocalization of Rhcg1 with NHE2 and NHE3b in PNA^+^ ionocytes of rainbow trout (Figure 5C). In European sea bass, *Rhcg2* mRNA expression is high in gills of freshwater compared to seawater acclimated fish (Blondeau-Bidet et al., 2019), but there is no information about Rhcg1 subcellular localization in ionocytes.

There is also considerable support for apical Na^+^ uptake through NHE2 or NHE3 with data from numerous teleost fish species of freshwater and marine origins, with species-specific differences (Figure 5). In several non-model euryhaline species, such as the Japanese sea bass (Inokuchi et al., 2017), European sea bass (Blondeau-Bidet et al., 2019), and Mozambique tilapia (Hiroi et al., 2005; Inokuchi et al., 2008), NHE3 is apically localized in ionocytes that express basolateral NKA (Figure 5A). Euryhaline species that move naturally between marine and freshwater habitats must be able to switch the function of their branchial epithelium between excretory and absorptive roles. There is increasing evidence that during freshwater acclimation, seawater-type ionocytes expressing apical NHE3 and high levels of basolateral NKCC1 are able to differentiate into freshwater-type ionocytes expressing high levels of NHE3 and decreasing levels of NKCC1 (Hiroi et al., 2005; Inokuchi et al., 2008) (Figure 5A). This plasticity of ionocytes to shift from an ion excreting to an ion absorptive function is remarkable and has been observed in only a few non-model species that are considered to be truly euryhaline (Inokuchi et al., 2017; Blondeau-Bidet et al., 2019). Thus, NHE3 seems essential in both seawater and freshwater environments for H^+^ excretion. The presence of NHE-type cells could be advantageous for species facing environmental challenges, such as fluctuating environmental CO_2_ (Montgomery et al., 2022). Determining the expression patterns of cooperating transporters that potentially form a functional Na^+^/NH_4_^+^exchange complex (see section above) in this NHE3-type cell would help us understand the role of NHE2 and NHE3 in effective Na^+^ uptake. This finding would suggest the presence of ammonia excretion and provides additional evidence for Model 2.

#### Support for model 3 in teleost fish

In teleost fish, there is evidence for coupled NaCl uptake mediated by an apical Na^+^, Cl^−^ cotransporter (NCC2 [or NCC-like], SLC12A10 in gills; NCC1, SLC12A3 in kidney), providing support for Model 3 (Figure 1C) (Hiroi et al., 2008; Wang et al., 2009; Blondeau-Bidet et al., 2019; Breves et al., 2021). This mechanism is contrary to earlier assumptions of independent Na^+^ and Cl^−^ transport across fish gills (Krogh, 1938). Ionocytes with apical (in fresh water) and basolateral (in seawater) NKCC/NCC immunolocalization were first identified in Mozambique tilapia (Wu et al., 2003), using a heterologous antibody that recognizes NKCC1, NKCC2, and NCC cotransporters (Wu et al., 2003). It is likely that this antibody recognized apical NCC2 rather than an apical NKCC2 in fish gills. This localization of NCC/NKCC was then confirmed in several additional species (see review by Hiroi and McCormick, 2012 and sections above). Apical NCC2 is in fact highly expressed in gills of freshwater-acclimated fish, rather than NKCC2, which is expressed in intestine and kidney (review in Hiroi and McCormick, 2012). In apical NCC2 expressing ionocytes in fish gills, basolateral NKA and NBC1 are thought to facilitate Na^+^ extrusion to the blood (Hiroi et al., 2005; Hwang, 2009) and a basolateral chloride channel (potentially ClC2/3) is involved in chloride export to the blood (Tang and Lee, 2011; Bossus et al., 2013). NKA-positive cells with apical NCC2 (called type II ionocytes in tilapia, corresponding to Model 3, Figure 1C) have only been identified in strictly freshwater fish or euryhaline freshwater-acclimatized fish, and are thus clearly identified as freshwater-type ionocytes (Hiroi et al., 2008; Inokuchi et al., 2008, 2017; Hsu et al., 2014; Blondeau-Bidet et al., 2019).

When euryhaline fish are transferred from seawater to fresh water, NCC-type cells are newly synthesized, within 7–14 days of acclimation (Inokuchi et al., 2017; Blondeau-Bidet et al., 2019). Their localization within the gill epithelium (lamellar or filamentary) can differ from NHE3-type cells, as is the case for Japanese and European sea bass (Inokuchi et al., 2017; Blondeau-Bidet et al., 2019). NHE3-type cells (Model 2, Figure 1B), on the contrary, exist in freshwater and seawater-acclimated fish due to their dual roles of ion uptake and acid secretion (Dymowska et al., 2012).

NCC-type ionocytes are not present in all teleost fish species. For instance, the absence of NCC-type ionocytes in several diadromous species (salmonids, eels) is intriguing and requires further investigation. NCC-type cells are also absent in gills of seawater-acclimated teleost fish.

NCC cells (Model 3, Figure 1C) are essential for NaCl uptake in freshwater environments for certain fish species, particularly under low Cl^−^ and/or potentially low pH conditions. In Mozambique tilapia maintained under low Cl^−^ or a combination of low Cl^−^ and low Na^+^, the apical surface of NCC cells is much larger than in control fish (Inokuchi et al., 2009). These morphological changes at the cellular level suggest an important role of NCC cells in Cl^−^ poor environments. Gill mRNA expression of zebrafish *NCC2* (*SLC12a10.2*) and Mozambique tilapia *NCC* (the NCC paralog was not indicated) (Inokuchi et al., 2009) was induced by a low-Cl^-^ environment, but not by a low-Na^+^ environment (Inokuchi et al., 2009; Wang et al., 2009). Also, NCC protein expression was induced in low Cl^−^ conditions, suggesting an important role in Cl^−^ uptake. Finally, following acute acidosis (low pH), whole body Na^+^ and Cl^−^ content decreased in larval zebrafish, indicating that low pH induces ion loss and temporary inhibition of Na^+^ uptake (Kwong and Perry, 2016). In these fish, compensatory Na^+^ and Cl^−^ uptake resulted exclusively from increased function of NCC-cells (Kwong and Perry, 2016). Studies in additional species are necessary to determine the functional role of NCC in Na^+^ uptake in acidic environments.

In addition, NCC functions are redundant with NHE3 for Na^+^ uptake in certain fish. But, as mentioned above, NCC function is favored under low Cl^−^ and/or potentially low pH conditions, whereas NHE3 is not. Na^+^ uptake *via* NHE3 is more challenging in an acidic environment, notably in the absence of interaction with Rh proteins (see Model 2). In zebrafish larvae, knockdown of *NHE3* caused an increase in NCC-expressing ionocytes, whereas knockdown of *NCC* increased the number of NHE3-expressing cells (HR cells) (Wang et al., 2009; Chang et al., 2013). In each knockdown experiment, whole-animal Na^+^ uptake was recovered due to the compensatory regulation of either NCC or NHE3. The presence of both Na^+^ uptake mechanisms in some fish gill epithelia (Models 2 and 3) enables them to maintain whole-animal Na^+^ homeostasis under different environmental conditions, depending on water pH (Hirata et al., 2003), ionic strength, and potentially other factors, such as water hardness (Wood et al., 1998; Wilson et al., 1999) and possibly temperature (Kwong et al., 2014).

Interestingly, gills of the killifish *Fundulus heteroclitus* are known to possess only one ionocyte subtype, with apical NCC and NHE and basolateral VHA coexpressed with NKA (Katoh et al., 2003; Breves et al., 2020). The basal localization of VHA with NKA, based on *in situ* immunolocalization, suggests an alternative Na^+^ uptake mechanism in this species (Katoh et al., 2003). This configuration of apical NCC and NHE (Katoh et al., 2003; Breves et al., 2020) differs drastically from other species, where NHE and NCC are expressed in different cell types (see the sections above). Intriguingly, in killifish no discernible Cl^−^ uptake has been measured in gills, suggesting that NCC is involved in Na^+^ uptake, but not Cl^−^ uptake. Acclimation from brackish to freshwater conditions increases the mRNA expression of *carbonic anhydrase 2 (CA2), VHA, Na^+^/H^+^ exchanger 2 (NHE2)*, as well as *Na^+^,HCO_3_^−^ cotransporter 1 (NBC1)* (Scott et al., 2005). These results suggest that both basolateral pumps VHA and NKA would generate an electrical gradient (negatively charged cells) driving apical Na^+^ through NHE-like proteins (possibly NHE2, Edwards et al., 2005) or NCC (see above; Dymowska et al., 2012). More functional data are required to validate this alternative model, in which basolateral VHA is involved in Na^+^ uptake.

### Conclusions on ion uptake in teleost fish, focusing on Na^+^ uptake

A striking feature of ion uptake mechanisms in teleost fish is the diversity of ionocyte types and the variety of configurations of ion transporters within ionocytes. There is evidence for the presence of all three hypothesized classes of models (Figure 1) in teleost fish. Several studies have shown that environmental conditions, such as pH, Na^+^, and Cl^−^ levels, are key factors that determine which Na^+^ uptake model is more prevalent. However, we have no understanding of whether there is a phylogenetic pattern in the occurrence of these models among taxa. Too few fish models have been studied in detail to determine whether more closely related fish have more similar mechanisms of ion uptake.

The understanding of ion uptake mechanisms in fish gills have been enhanced by the availability of fish genomes and complementary approaches to identify the presence and function of ion transporters (i.e.*,* non-invasive scanning ion-selective electrode technology (SIET) to measure ion transport at the cellular level). Novel molecular approaches such as single-cell RNA sequencing would help identify new ionocyte cell types and elucidate ion transport mechanisms.

Relative to insects and crustaceans, an additional model (Model 3) of ion uptake is available in some fish species, notably involving the NCC-like (or NCC2) cotransporter in gills (Hiroi and McCormick, 2012). This NCC-like cotransporter provides some evidence for coupled NaCl uptake, rather than the canonical independent transport of Na^+^ and Cl^−^. This type of cotransport had previously been demonstrated in mammalian renal distal convoluted tubules (Jacquillet et al., 2011).

Substantial evidence supports the presence of Model 2 involving NHE2 or NHE3 along with Rh proteins working together as a metabolon. NHE3 or NHE2-type cells are observed in numerous fish species. However, whether Rh proteins are also present in these cell types remains to be investigated in non-model fish species.

There is still some debate regarding the unknown Na^+^ uptake mechanisms in fish to support Model 1 (Figure 1A), notably in harsh environments, such as very low pH. Recently, Clifford et al. (2022) has identified another K^+^-dependent Na^+^ uptake mechanism at low pH in zebrafish, but more evidence is necessary for this mechanism in other teleost species. Also, ASICs have been investigated only in a few teleost species and need to be further explored in non-model species. No evidence is available for the role of an NHA antiporter involved in Na^+^ uptake, despite the presence of *NHA* in vertebrate genomes, including in teleost fish (Brett et al., 2005). The potential involvement of NHA in gills and other osmoregulatory organs should be investigated, notably in renal and intestinal epithelial cells. In addition, there has been much less effort devoted to investigating Na^+^ re-uptake mechanisms in the fish kidney, which is a main organ contributing to Na^+^ homeostasis in fish.

VHA is mainly localized apically in fish gill ionocytes. However, in numerous non-model fish species, VHA has not yet been localized. The presence of basolateral VHA in gill ionocytes of killifish is intriguing and requires further investigation. Ionocytes with basolateral VHA potentially provide alternative models for Na^+^ uptake in species that have been less extensively investigated thus far.

## Concluding remarks on Na^+^ uptake in aquatic organisms

Studies support sharply different models of ion uptake from fresh water between crustaceans/insects and teleost fish. However, it is still premature to make strong conclusions on the details of these models and their prevalence within and between these groups. We have not sampled enough taxa to know how widespread each model is among taxa and whether there is a phylogenetic pattern in their occurrence. For both arthropods and teleost fish, information on mechanisms of ion transport is completely lacking for many (if not most) lineages.

As of yet, ionocyte models for crustaceans are often hypothetical, where functional studies are often not linked to molecular evidence of specific ion transporters. For crustaceans and insects living in very dilute environments, available physiological, molecular, and histological data show predominant support for Model 1 (Figure 1A), or more complex variants of this model involving one or two cells (Figures 2, 3). In all models, the main driving force for ion transport is provided by the apical VHA and the basolateral NKA.

However, sufficient data are lacking to make any strong conclusions involving the sodium transporters involved. In particular, a major problem is that functional analyses do not exist in crustaceans for the top candidate Na^+^ transporter, the Na^+^/H^+^ antiporter (NHA), while only a few studies exist for insects (e.g., Xiang et al., 2012; Chintapalli et al., 2015). Also, it is not clear in crustacean models whether different Na^+^ uptake models operate under different environmental conditions (as is the case for teleost fish). Future studies should focus on analyzing the stoichiometry of ion transport for NHA paralogs in crustaceans and aquatic insects. In addition, the presence and roles of NCC proteins or ASICs in arthropod taxa still require investigation.

In teleost fish, there is some support for all three models of ion uptake from fresh water (Figure 1) and some data suggest the presence of novel and unique models of Na^+^ uptake. In most teleost species, more than one ionocyte subtype has been identified in gills, with up to five ionocytes subtypes occurring in zebrafish. The prevalence of a particular Na^+^ uptake pathway in gills or skin depends on environmental conditions, such as pH, Na^+^, or Cl^−^ levels. Future studies should also focus on non-model teleost species to gain a better understanding of the diversity of ion uptake mechanisms in fish. Our understanding of ion uptake mechanisms in gills needs to be completed in conjunction with studies on ion uptake mechanisms in other osmoregulatory organs, notably the kidney (Takvam et al., 2021).

Whole-genome analyses have the potential to reveal novel ion transporter gene families and paralogs across a wide variety of taxa. However, analyses of ion transporter gene families are still in their infancy for most crustacean taxa, even as more crustacean genomes are becoming available. Genomic studies of the copepod *E. affinis* complex have been valuable in revealing strong selection acting on the *NHA* gene family, as well as other ion transporter families, and implicating NHA paralogs in freshwater adaptation (Posavi et al., 2020; Stern and Lee, 2020; Stern et al., 2022). Single-cell RNA sequencing could greatly improve our knowledge on the diverse ionocyte subtypes in fish and would potentially help identify new Na^+^ uptake pathways in specific cell types (Leguen et al., 2015; Xue et al., 2015). As more genomes become available for non-model species, comparative genomic studies could facilitate invaluable discoveries on novel and diverse ion transport systems.

At this point, relatively little cross communication occurs among investigators working on ion transport mechanisms in different taxa (e.g., crustaceans versus fish). A key problem is that the nomenclature for ion transporters varies wildly across taxa, causing much confusion on which ion transporters are being studied and which ion transporters are homologous between different taxa. Performing many more phylogenetic studies of ion transporter gene families that include a broad range of taxa, including arthropods and vertebrates, would be helpful in determining the homology of ion transporters across taxa. Such clarification of nomenclature and homologous relationships of ion transporter gene families will make it far easier to compare results from studies across a broader range of taxonomic groups, broadening our inferences on the prevalence and peculiarities of ion transport mechanisms in nature.
